# Nerve Growth Factor Signaling Promotes Nuclear Translocation of TRAF4 to Enhance Tumor Stemness and Metastatic Dormancy Via C‐Jun‐mediated IL‐8 Autocrine

**DOI:** 10.1002/advs.202414437

**Published:** 2024-12-24

**Authors:** Kai Zhao, Tifan Sun, Qiruo Sun, Zhenzhong Chen, Tiepeng Wang, Jinming Yang, Lei Li, Yanan Zhu, Xinye Liu, Dawei Yang, Binyan Lin, Na Lu

**Affiliations:** ^1^ State Key Laboratory of Natural Medicines Jiangsu Key Laboratory of Carcinogenesis and Intervention Department of Physiology School of Basic Medicine and Clinical Pharmacy China Pharmaceutical University 24 Tongjiaxiang Nanjing 210009 China; ^2^ School of Pharmacy Nanjing University of Chinese Medicine 138 Xianlin Rd. Nanjing 210023 China; ^3^ Department of Pharmacy The Second Hospital of Nanjing Affiliated Hospital to Nanjing University of Chinese Medicine Nanjing 210003 China; ^4^ Department of General Surgery The First Affiliated Hospital of Nanjing Medical University Nanjing 210029 China

**Keywords:** IL‐8, metastatic dormancy, nuclear translocation, stemness, TRAF4

## Abstract

Tumor necrosis factor receptor‐associated factor 4 (TRAF4), an E3 ubiquitin ligase, is frequently overexpressed in tumors. Although its cytoplasmic role in tumor progression is well‐documented, the precise mechanisms underlying its nuclear localization and functional contributions in tumor cells remain elusive. This study demonstrated a positive correlation between the expression of nuclear TRAF4 and both tumor grades and stemness signatures in human cancer tissues. Notably, reduced nuclear TRAF4 led to decreased stemness properties and metastatic dormancy of tumor cells. Conversely, restoring nuclear TRAF4 in TRAF4‐knockout (TRAF4‐KO) cells augmented these cellular capabilities. Within the nucleus, the TRAF domain of TRAF4 interacted with c‐Jun, thereby stimulating its transcriptional activity. This interaction subsequently led to an enhancement of the promoter activity of interleukin‐8 (IL‐8), which is identified as a mediator of nuclear TRAF4‐induced tumor dormancy. Additionally, activation of AKT signaling by nerve growth factor facilitated TRAF4 phosphorylation at Ser242, enhancing its interaction with 14‐3‐3θ and promoting its nuclear translocation. Importantly, pharmacological modulation of TRAF4 nuclear translocation is found to suppress tumor tumorigenicity and metastasis in tumor models. This study highlights the critical role of nuclear TRAF4 in regulating tumor stemness and dormancy, positioning it as a potential therapeutic target for metastatic and refractory cancers.

## Graphical abstract

TRAF4, an E3 ubiquitin ligase, is frequently overexpressed in tumors, and its nuclear localization is correlated to tumor grade and stemness. Reduced nuclear TRAF4 decreased stemness and metastasis, while its restoration enhanced these traits. TRAF4 interacted with c‐Jun to activate IL‐8, promoting dormancy. Additionally, AKT signaling facilitated TRAF4 phosphorylation, aiding nuclear translocation, and making it a potential target for cancer therapy.

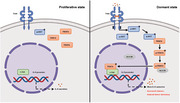

## 1 Introduction

Despite significant advancements in surgical resection techniques and systemic therapies for primary tumors, metastasis remains the primary cause of mortality in over 90% of cancer patients.^[^ ^1^ ^]^ The incurability of metastatic tumors stems from the presence of numerous micrometastases^[^ ^2^ ^]^ and their resistance to conventional therapeutic strategies.^[^ ^3^, ^4^ ^]^ The process of tumor metastasis encompasses a complex series of interconnected steps.^[^ ^5^, ^6^ ^]^ First, tumor cells undergo epithelial‐mesenchymal transition (EMT), a process that endows them with an invasive phenotype.^[^ ^5^ ^]^ This newly acquired phenotype facilitates the secretion of matrix metalloproteinases (MMPs),^[^ ^7^ ^]^ which enable the cells to invade the adjacent tissues and subsequently enter the circulatory system. Once in circulation, the viable tumor cells adhere to and penetrate the vessel walls, eventually establishing metastatic dormancy within the target organ.^[^ ^8^ ^]^ The dormancy phase of metastatic cells can persist for extended periods, ranging from months to years, or even decades, without clinical detection. This latent state can ultimately lead to tumor recurrence upon activation.^[^ ^9^, ^10^ ^]^ Recent studies have revealed that the initial stages of metastasis are highly efficient, whereas the later stages are relatively inefficient, highlighting the potential of targeting metastatic dormancy as a promising therapeutic strategy.^[^ ^11^, ^12^, ^13^ ^]^ Despite its significance, the underlying mechanisms that regulate metastatic dormancy remain poorly understood.

Cancer stem cells (CSCs) represent a subpopulation of cancer cells that can produce a variety of heterogeneous tumor cells and drive the initiation and progression of tumors.^[^ ^14^, ^15^, ^16^ ^]^ These cells often exist in a dormant state and may play a crucial role in metastatic dormancy due to their profound resistant to the majority of chemotherapeutic agents.^[^ ^17^, ^18^ ^]^ Evidence suggests that within a tumor cell population, any cell may exhibit stem‐like properties owing to the dynamic and plastic nature of stemness in tumor cells.^[^ ^12^, ^19^, ^20^ ^]^ This process shares similarities with epithelial‐mesenchymal transition (EMT).^[^ ^21^ ^]^ Cancer cells undergoing EMT demonstrate an augmentation in CSC‐like traits and anti‐apoptotic capabilities, rendering them more likely to succeed in metastasis.^[^ ^22^, ^23^ ^]^ Consistent with the self‐renewal ability of CSCs in tumor initiation,^[^ ^24^ ^]^ tumor dormancy^[^ ^25^ ^]^ can also emanate from a very limited number of tumor cells and is pertinent to delayed cancer recurrence.^[^ ^26^ ^]^ Furthermore, both CSCs and dormant tumor cells exhibit resistance to anti‐tumor immune responses and can be re‐activated by microenvironmental cues, such as angiogenesis.^[^ ^27^, ^28^ ^]^ A single dormant cancer cell has the potential to develop into macrometastases through increased secretion of angiogenic factors in the metastatic niche.^[^ ^29^ ^]^ Similarly, it fosters tumor angiogenesis by secreting vascular endothelial growth factor (VEGF).^[^ ^30^, ^31^ ^]^ Consequently, there exist notable parallels between the theories pertaining to CSCs, EMT, and metastatic dormancy.

Tumor necrosis factor receptor‐associated factor 4 (TRAF4) is a member of the TRAF protein family.^[^ ^32^ ^]^ It acts as an adaptor protein with the capability to bind directly to the members of tumor necrosis factor superfamily and Toll‐like receptor, thereby initiating downstream signaling cascades. In tumors, gene amplification represents the most frequently observed genetic alteration involving TRAF4.^[^ ^33^ ^]^ Additionally, TRAF4 shows abnormally elevated expression levels in 43% of tumor tissues across various known types of human cancers,^[^ ^34^, ^35^, ^36^, ^37^ ^]^ encompassing breast cancer, endometrial cancer, and prostate cancer. Within the cytoplasm, TRAF4 frequently functions as an E3 ubiquitin ligase, regulating the activation of diverse kinases, such as AKT1 and TAK1,^[^ ^37^, ^38^ ^]^ through the promotion of ubiquitination processes. As a result, TRAF4 can accelerate tumorigenesis,^[^ ^39^ ^]^ metastasis,^[^ ^36^, ^37^ ^]^ and therapeutic resistance.^[^ ^34^, ^35^ ^]^ Our recent studies have elucidated that TRAF4 also plays a crucial role in glioblastoma by driving stemness and temozolomide resistance.^[^ ^34^ ^]^ The underlying mechanism through which this occurs entails TRAF4 functioning as a scaffolding protein to facilitate USP7‐mediated deubiquitination, independently of its proteolytic catalytic activity. These findings demonstrate the pivotal role of TRAF4 in tumor initiation and progression, indicating it as a critical target for cancer therapeutic interventions.

In addition to its cytoplasmic effect, TRAF4 possesses two nuclear localization signals, which are distinctive features within the TRAF family.^[^ ^32^, ^40^ ^]^ Despite the observable nuclear localization of TRAF4 in tumor cells and tissues, the clinical implications of this phenomenon have yet to be reported. Furthermore, the precise molecular mechanism underlying the nuclear transport, especially its upstream signaling pathways and function in the nucleus, remains unclear. Here, we uncovered a robust clinical correlation between nuclear TRAF4 and tumor grades. Initiates a cascade that activates AKT, leading to the phosphorylation of TRAF4 at Ser242. Subsequently, phosphorylated TRAF4 binds to 14‐3‐3θ, facilitating its translocation into the nucleus. Within the nucleus, TRAF4 interacts with the transcription factor c‐Jun, thereby promoting the transcription of interleukin‐8 (IL‐8) during metastatic dormancy. Finally, we have identified a natural product capable of inhibiting the nuclear translocation of TRAF4 from the plasma membrane, presenting a novel and promising therapeutic approach for the treatment of metastatic cancer.

## 2 Results

### 2.1 Nuclear Accumulation of TRAF4 Positively Correlates with Tumor Grade and Metastatic Potential

**Figure 1:**
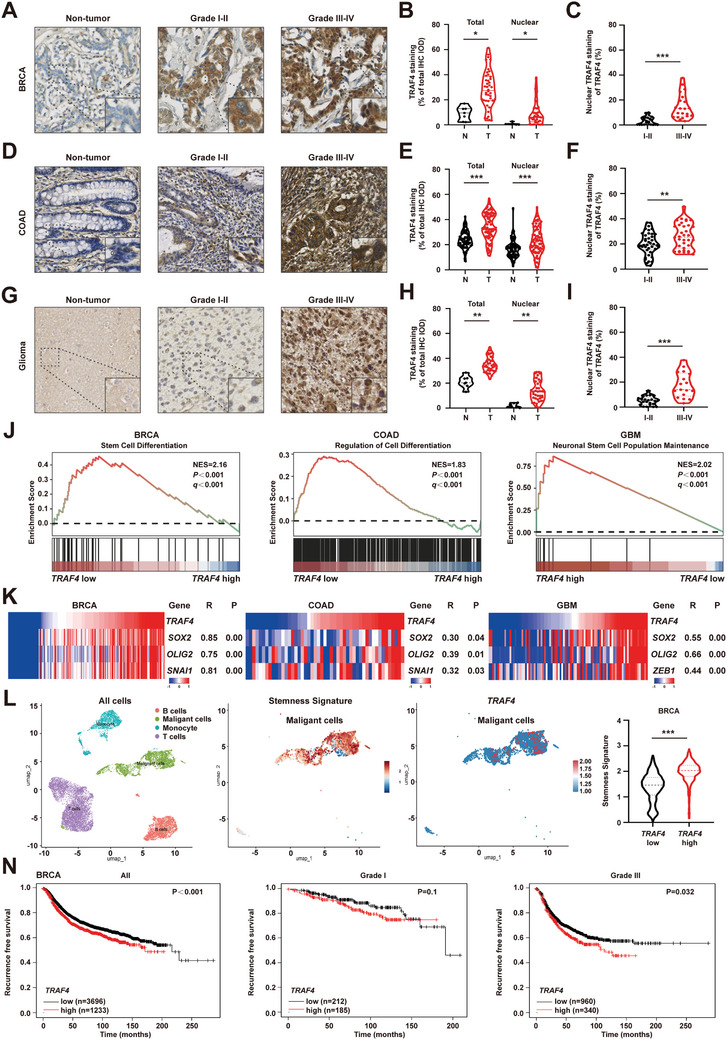
Nuclear Accumulation of TRAF4 Positively Correlates with Tumor Grade and Metastatic Potential. A) Representative IHC images (magnification × 10, and × 20) of the TRAF4 staining in a microarray consisting of BRCA tissues (n = 42). BRCA, breast cancer; Bar, 100 µm. B) Nuclear localization of TRAF4 is significantly increased in BRCA tissues. Quantification of total TRAF4 and nuclear TRAF4 in BRCA tissues from the microarray is shown. Staining (%) is calculated using Image J by comparing total and nuclear TRAF4 IHC. The integrated optical density (IOD) with total IOD values were extracted from the image. C) Nuclear TRAF4 level (%) is positively correlated with tumor grade in BRCA patients. D) Representative IHC images (magnification × 10, and × 20) of TRAF4 staining in a microarray consisting of COAD tissues (n = 74). COAD, colon adenocarcinoma; Bar, 100 µm. E) Nuclear localization of TRAF4 is significantly increased in COAD tissues. Quantification of total TRAF4 and nuclear TRAF4 in COAD tissues from the microarray is shown. Staining (%) is calculated using Image J by comparing total and nuclear TRAF4 IHC. The IOD with total IOD values were extracted from the image. F) Nuclear TRAF4 level (%) is positively correlated with TNM stages in COAD tissues. G) Representative IHC images (magnification × 10, and × 20) of TRAF4 staining in a microarray consisting of glioma tissues (n = 39). Bar, 100 µm. H) Nuclear localization of TRAF4 is significantly increased in glioma tissues. Quantification of total TRAF4 and nuclear TRAF4 in glioma tissues from the microarray is shown. Staining (%) is calculated using Image J by comparing total and nuclear TRAF4 IHC. The IOD with total IOD values were extracted from the image. I) Nuclear TRAF4 level (%) is positively correlated with TNM stages in glioma patients. J) The samples of GEO DataSets (GSE17907, GSE23806, GSE24514) were divided into samples with high TRAF4 expression level and samples with low TRAF4 expression level; grouped samples were analyzed by GSEA. K) Heatmap of GEO DataSets (GSE17907, GSE23806, GSE24514) of samples with high TRAF4 expression level and samples with low TRAF4 expression level, showing the expression of SOX2, OLIG2 and SNAIL1 in samples with high TRAF4 expression level compared with samples with low TRAF4 expression level. L) Visualization of TRAF4 high and low cells in BRCA tissue by UMAP (The samples of GEO DataSets, GSE148673). Visualization of TRAF4 and stemness signature genes expression (Runx1, Sox4, Sox9, Klf4, Klf9, Lgr4, Pou6f1) in BRCA tissue by UMAP. Violin plot showing expression levels of stemness signature in TRAF4 high and low cells. The tips of the violin plot represent minima and maxima, and the width of the violin plot shows the frequency distribution of data. N) Kaplan–Meier analysis of the association between recurrence‐free survival and TRAF4 expression in patients with BRCA. The analyses for all BRCA patients (left), Grade I BRCA patients (middle) and Grade III BRCA patients (right) are shown. Statistical significance between two groups was determined with two tailed Student's *t*‐test. Statistical significance among groups was determined by one‐way ANOVA test. Data are presented as mean ± SD. ^*^ *p* < 0.05, ^**^ *p* < 0.01, ^***^ *p* < 0.001, n.s indicates non‐significant.

To ascertain that nuclear localization of TRAF4 is not an artifact resulting from overexpression, we examined endogenous nuclear TRAF4 expression across various cancer types (Figure S1A–D, Supporting Information). Compared with non‐tumor cells, nuclear TRAF4 levels were significantly elevated in multiple tumor cell lines, suggesting that the nuclear localization of TRAF4 is a general phenomenon. To assess the clinical relevance of TRAF4 nuclear translocation, we performed immunohistochemical staining (IHC) analyses on tissue microarrays derived from human breast cancer (**Figure** **1**A–C), colon cancer (Figure 1D–F), and glioma (Figure 1G–I). The results demonstrated a marked increase in nuclear TRAF4 expression within these tumor tissues compared with their corresponding normal counterparts. This finding was further corroborated by the western blotting assay, which revealed upregulated nuclear TRAF4 expression in breast tumor tissues (Figure S1E, Supporting Information). Notably, we observed a positive correlation between nuclear TRAF4 levels and tumor grades, suggesting that the translocation of TRAF4 into the nucleus plays a critical role in tumor aggressiveness and differentiation status. To illustrate the impact of TRAF4's nuclear translocation on tumor development, MMTV‐PyMT mice were utilized for our studies; we found that nuclear expression of TRAF4 was exclusively present in PyMT tumors and absent in normal mammary tissues (Figure S1F,G, Supporting Information). Furthermore, its expression increased with advancing tumor progression (Figure S1H, Supporting Information). Cell lines derived from MDA‐MB‐231 and 4T1 exhibiting enhanced metastatic capabilities also displayed elevated levels of nuclear TRAF4 expression (Figure S1I, Supporting Information). To identify potential downstream pathways influenced by TRFA's accumulation within the nucleus, we performed Gene Set Enrichment Analysis (GSEA) using data from The Cancer Genome Atlas (TCGA) dataset; this analysis indicated that higher levels of TRFA correlated negatively with cellular differentiation while positively correlating with stem cell maintenance characteristics (Figure 1J). Additionally, there was a significant correlation between TRFA and numerous genes associated with stemness as well as epithelial‐mesenchymal transition processes such as OLIG2 (Figure 1K; Figure S1J, Supporting Information). Subsequently, single‐cell RNA sequencing data analysis confirmed an enrichment signature indicative of stemness among high‐TRAF‐high expressing tumoral cells (Figure 1L; Figure S1K, Supporting Information). To elucidate the implications of *TRAF4* in clinical outcomes, a Kaplan‐Meier plotter survival analysis was performed. The results revealed that high *TRAF4* expression strongly correlated with recurrence‐free survival (Figure 1N), especially in populations characterized by high‐grade or lymph metastasis populations (Figure S1L–N, Supporting Information), suggesting a poor prognostic value attributed to the elevated expression of TRAF4. These data indicated a potential correlation between the nuclear accumulation of TRAF4 and the differentiation degree or metastatic potential of cancer cells.

### 2.2 Inhibition of TRAF4 Nuclear Translocation Suppresses Tumor Stemness and Blocks Metastatic Dormancy

**Figure 2:**
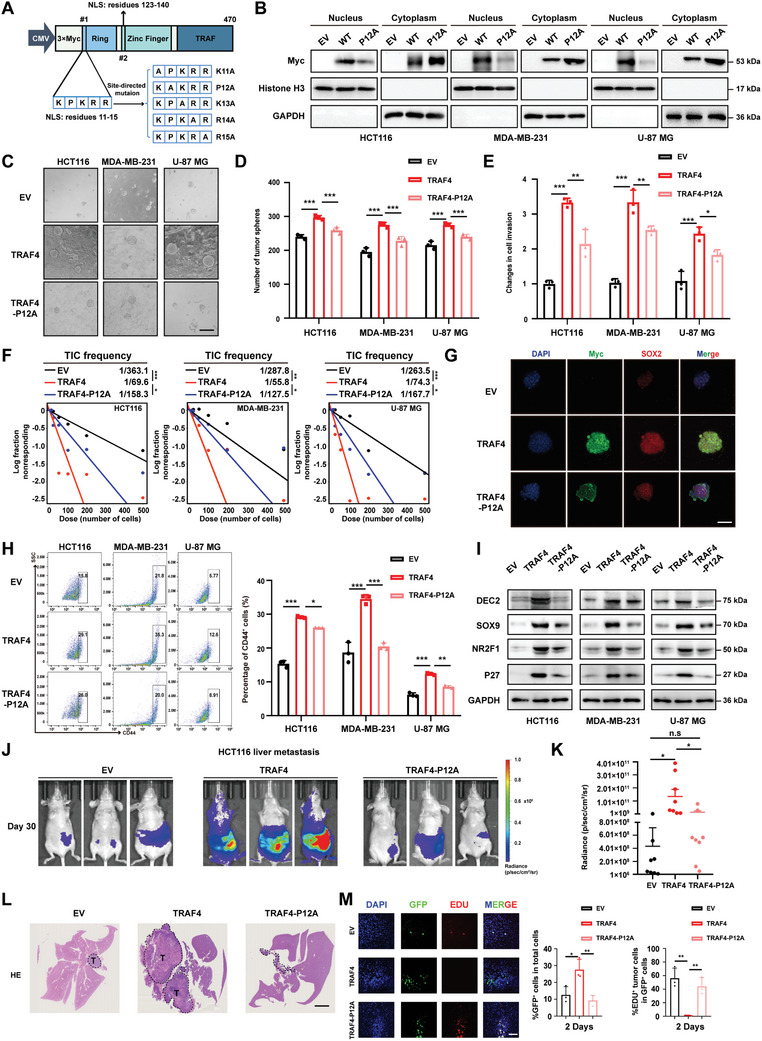
Abolished TRAF4 nuclear translocation inhibits tumor stemness and metastatic dormancy. A) Construction of TRAF4 plasmid with mutated nuclear localization sequence at 11–15 amino acids. B) EV, TRAF4 or TRAF4‐P12A were expressed in HCT116, MDA‐MB‐231 and U‐87 MG cells. C,D) Representative images of tumorspheres and their number quantitation of HCT116, MDA‐MB‐231 and U‐87 MG cells overexpressing EV, TRAF4 or TRAF4‐P12A. Scale bar, 25 µm. E) The quantification of cell invasion of HCT116, MDA‐MB‐231, U‐87 MG overexpressing EV, TRAF4 or TRAF4‐P12A. F) Frequency of tumor sphere formation was tested by in vitro limiting dilution assay in HCT116, MDA‐MB‐231, U‐87 MG overexpressing EV, TRAF4 or TRAF4‐P12A. G) IF images of expression of Myc‐TRAF4 or Myc‐TRAF4‐P12A (green) and SOX2(red) in HCT116 tumorsphere. Scale bar, 150 µm. H) CD44 expression was detected in HCT116, MDA‐MB‐231 and U‐87 MG cells overexpressing EV, TRAF4 or TRAF4‐P12A. Right side is its quantification. I) Immunoblotting analysis of DEC2, NR2F1, SOX9, p27and GAPDH in HCT116, MDA‐MB‐231 and U‐87 MG cells overexpressing EV, TRAF4 or TRAF4‐P12A. J,K) A total of 2 × 10^6^ HCT116 cells overexpressing EV, TRAF4 or TRAF4‐P12A were injected into the spleen to establish the colorectal cancer liver metastasis model. After 30 days, Bioluminescence was detected (J). Quantification of Bioluminescence (K) was shown. L)Representative images of H&E staining from these groups of mice. Scale bar, 10 µm. M) Seeding and proliferation of HCT116 overexpressing EV, TRAF4 or TRAF4‐P12A subpopulations in livers at day 2 after the injection into the spleen Shown are representative immunofluorescences (IF) images, a percentage of GFP^+^ tumor cells in total cells (middle) and EdU^+^ proportions of tumor cells (right); *n*  =  RMFs from 3 mice. Scale bar, 150 µm. Each experiment was performed at least three times. Statistical significance between two groups was determined with two tailed Student's *t*‐test. Statistical significance among groups was determined by one‐way ANOVA test. Data are presented as mean ± SD. ^*^ *p* < 0.05, ^**^ *p* < 0.01, ^***^ *p* < 0.001, n.s indicates non‐significant.

**Figure 3:**
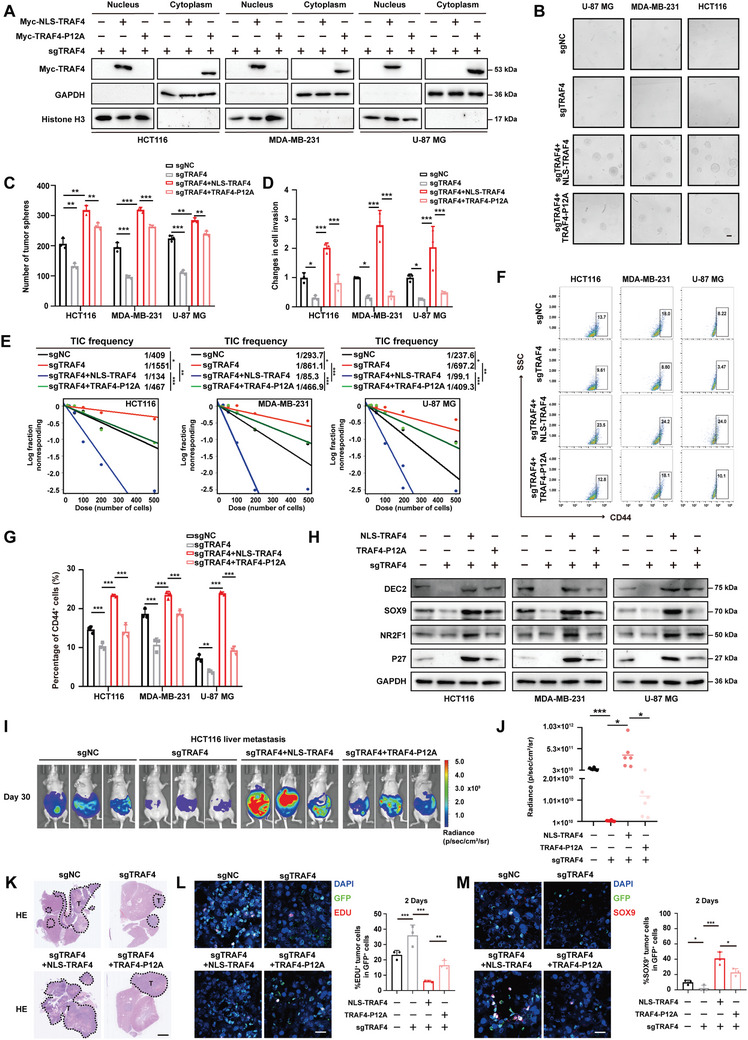
Elevated Nuclear Accumulation of TRAF4 Enhances Tumor Stemness and Induces Metastatic Dormancy. A) EV, NLS‐TRAF4 or TRAF4‐P12A were expressed in TRAF4‐KO HCT116, MDA‐MB‐231 and U‐87 MG cells. Nuclear plasma separation experiment was conducted and the expression of Myc‐TRAF4 was analyzed by immunoblotting assay. GAPDH and Histone H3 were used as cytoplasmic and nuclear internal references, respectively B,C) Representative images of tumorspheres and their number quantitation of TRAF4‐KO HCT116, MDA‐MB‐231 and U‐87 MG cells overexpressing EV, NLS‐TRAF4 or TRAF4‐P12A. Scale bar, 25 µm. D) The quantification of cell invasion of TRAF4‐KO HCT116, MDA‐MB‐231 and U‐87 MG overexpressing EV, NLS‐TRAF4 or TRAF4‐P12A. E) Frequency of tumor sphere formation was tested by in vitro limiting dilution assay in TRAF4‐KO HCT116, MDA‐MB‐231 and U‐87 MG overexpressing EV, NLS‐TRAF4 or TRAF4‐P12A. F,G) CD44^+^ cell subpopulation and their quantification was tested by flow cytometry analysis in TRAF4‐KO HCT116, MDA‐MB‐231 and U‐87 MG overexpressing EV, NLS‐TRAF4 or TRAF4‐P12A. H) Immunoblotting analysis of DEC2, SOX9, p27 and NR2F1 and GAPDH in TRAF4‐KO HCT116, MDA‐MB‐231 and U‐87 MG overexpressing EV, NLS‐TRAF4 or TRAF4‐P12A. I,J) 2 × 10^6^ TRAF4‐KO HCT116 cells overexpressing EV, NLS‐TRAF4 or TRAF4‐P12A were injected into the spleen to establish the liver metastasis model of colon cancer. After 30 days, Bioluminescence was detected(I). Quantification of Bioluminescence (J) was shown. K)Representative images of H&E staining from the above four groups of mice. Scale bar, 10 µm. L) GFP‐labeled TRAF4‐KO HCT116 cells overexpressing EV, NLS‐TRAF4 or TRAF4‐P12A were injected into the spleen. 1 days later, EDU was intraperitoneally injected and livers were collected after 24 h. Representative IF images and its quantification exhibited a percentage of EdU^+^ proportions in GFP^+^ HCT116 cells; n  =  RMFs from 3 mice. Scale bar, 150 µm. M) 2 days after the injection of GFP‐labeled HCT116 cells into the spleen. Livers were collected and stained with SOX9. Representative IF images and its quantification was shown. Each experiment was performed at least three times. Statistical significance between two groups was determined with two tailed Student's *t*‐test. Statistical significance among groups was determined by one‐way ANOVA test. Data are presented as mean ± SD. ^*^ *p* < 0.05, ^**^ *p* < 0.01, ^***^ *p* < 0.001, n.s indicates non‐significant.

To elucidate the functional role of TRAF4 nuclear translocation, we concentrated on a segment of the nuclear localization sequence within TRAF4 and individually mutated its amino acids (**Figure** **2**A). Among the five amino acids, only the mutation of proline at position 12 to alanine significantly diminished TRAF4's ability to enter the nucleus in HEK‐293T cells (Figure S2A, Supporting Information). In tumor cells, TRAF4‐P12A also inhibited TRAF4 nuclear accumulation compared with wild‐type TRAF4 (wt‐TRAF4) (Figure 2B; Figure S2B, Supporting Information). We found that TRAF4 enhanced tumor sphere formation (Figure 2C,D) and cell invasion (**Figure** **3**E) in HCT116, MDA‐MB‐231, and U‐87 MG cells; conversely, TRAF4‐P12A suppressed these processes. The in vitro limiting dilution assay further demonstrated that reduced nuclear translocation of TRAF4 led to a decreased frequency of tumor spheres (Figure 2F). Additionally, we noted that attenuated nuclear accumulation of TRAF4 resulted in a reduction in both sphere volume and SOX2 expression when compared to cells overexpressing TRAF4 (Figure 2G). Furthermore, we found that TRAF4 promoted the CD44^+^ cell population, decreased TRAF4 nuclear translocation correspondingly reduced the population size (Figure 2H). Consistently, biomarkers associated with cell dormancy as well as stemness were upregulated in tumor cells expressing high levels of TRAF4; however, their expression, including DEC2, SOX9, NR2F1, and P27, declined in cells expressingTRAFA‐P12A (Figure 3I; Figure S2C, Supporting Information). To investigate the role of TRAF4's nuclear accumulation throughout the metastasis process, we injected HCT116 cells via the spleen to construct a liver metastasis model. In this model, TRAFA drove liver metastasis, whereas TRFA‐P12A reduced it (Figure 3J–L). Notably, TRAF4 fostered an increase in the number of GFP^+^ HCT116 cells with lower proliferation rates (EDU negative) seeded in the livers. However, upon injection of HCT116 cells expressing the TRAF4‐P12A mutant into the livers, the GFP^+^ HCT116 cells exhibited a significantly enhanced proliferative capacity (Figure 3M). These findings suggest that loss‐of‐function mutations inhibiting nuclear accumulation of TRAF4 suppress the stemness characteristics of tumor cells, impeding tumor metastatic potential and increasing the seeding of proliferative tumor cells in livers.

### 2.3 Elevated Nuclear Accumulation of TRAF4 Enhances Tumor Stemness and Induces Metastatic Dormancy

Next, we inserted the nuclear localization signal sequence (NLS) of the synthetic viral SV40 into the N‐terminal of TRAF4 (Figure S3A, Supporting Information). To examine its nuclear accessibility, we conducted Western blotting and immunofluorescence assays (Figures S3B and S2D, Supporting Information). Our results indicated that the addition of NLS further enhanced TRAF4 nuclear accumulation in tumor cells, compared with wt‐TRAF4. This increased nuclear translocation of TRAF4 significantly facilitated tumor sphere formation (Figure S3C,D, Supporting Information) and cell invasion (Figure S3E, Supporting Information) in HCT116, MDA‐MB‐231, and U‐87 MG cells. We also confirmed that enhanced nuclear translocation of TRAF4 increased the frequency of tumor spheres through an in vitro limiting dilution assay (Figure S3F, Supporting Information). This greater accumulation of TRAF4 in the nucleus resulted in a notable increase in sphere volume, accompanied by elevated SOX2 expression (Figure S3G, Supporting Information). Additionally, we observed a rise in the CD44^+^ cell population (Figure S3H, Supporting Information), a widely recognized biomarker of mesenchymal cells. Markers associated with dormancy and stemness were upregulated in tumor cells expressing NLS‐TRAF4 (Figure S3I,J, Supporting Information). In the liver metastasis model of HCT116 cells, our findings demonstrated that increased TRAF4 nuclear accumulation led to a more significant number and size of metastatic nodules in the livers (Figure S3K–M, Supporting Information). Notably, one day after inoculation into nude mice, we noted a higher number of GFP^+^ HCT116 cells initially present in the livers when TRAF4 nuclear translocation was enhanced (Figure S3M, Supporting Information).

To further investigate the functional role of TRAF4's nuclear translocation, we employed the CRISPR‐Cas9 system to knock out (KO) *TRAF4* in HCT116, MDA‐MB‐231, and U‐87 MG cells. Two different sgRNAs effectively eliminated endogenous TRAF4 expression in these tumor cells (Figure S2E, Supporting Information). Subsequently, we stably expressed NLS‐TRAF4 and TRAF4‐P12A in the TRAF4‐KO tumor cells to restore the nuclear or cytoplasmic expression of TRAF4 (Figure 3A). Compared with the TRAF4‐KO group, the restoration of nuclear TRAF4 remarkably promoted tumor sphere formation (Figure 3B,C) and cell invasion (Figure 3D) in HCT116, MDA‐MB‐231, and U‐87 MG cells. It also increased the frequency of tumor spheres (Figure 3E) and the CD44^+^ cell population (Figure 3F), indicating that nuclear TRAF4 may enhance tumor stemness. Although the recovery of cytoplasmic TRAF4 also boosted tumor stemness, its effect was less pronounced than that of nuclear TRAF4. Notably, nuclear TRAF4 elevated the expression of biomarkers associated with tumor dormancy and stemness, while cytoplasmic TRAF4 had a weaker effect (Figure 3H; Figure S2F, Supporting Information). In a liver metastasis model of colon cancer, the results showed that nuclear TRAF4 overexpression in TRAF4‐KO HCT116 cells resulted in the formation of more and larger metastatic nodules in livers compared with the TRAF4‐KO or cytoplasmic TRAF4 group (Figure 3J–L). Additionally, nuclear TRAF4 increased the proportion of non‐proliferative (EDU^−^) GFP^+^ cells and quiescent (SOX9^+^) GFP^+^ cells in livers (Figure 3K–M). These results indicated that nuclear TRAF4 had the capacity to attenuate proliferation and sustain a dormant state. Consequently, nuclear TRAF4 promoted the stemness and dormancy of tumor cells, thus assisting in the seeding of tumor cells in livers.

### 2.4 Nuclear Accumulation of TRAF4 Induces IL‐8 Transcription and Autocrine Signaling to Sustain the Tumor Stemness Phenotype

**Figure 4:**
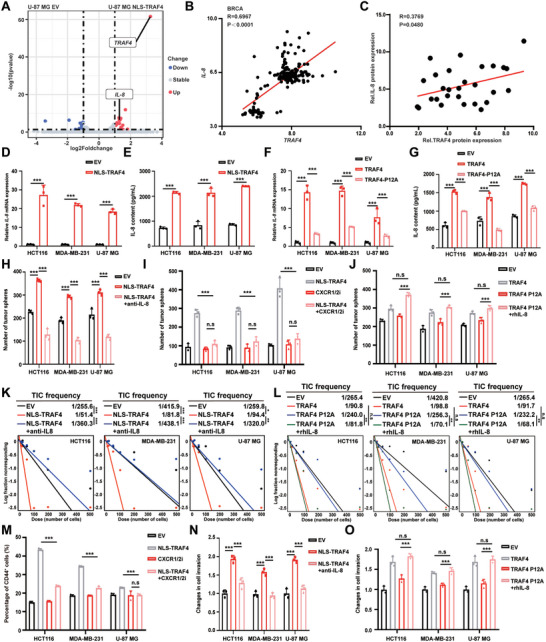
Nuclear Accumulation of TRAF4 Induces IL‐8 Transcription and Autocrine Signaling to Sustain the Tumor Stemness Phenotype. A) Schematic diagram for screening the upregulated candidate genes (q < 0.05, Log_2_FC > ) in U‐87 MG NLS‐TRAF4 cells compared to U‐87 MG EV cells and RNA‐sequencing was employed to identify differentially expressed genes in three pairs of U‐87 MG NLS‐TRAF4 cells and U‐87 MG EV cells. B) Correlation between *TRAF4* and *IL‐8* expression in BRCA tissues by using GEO dataset of GSE2990. C) Correlation between TRAF4 and IL‐8 expression in BRCA tissues by using immunoblotting analysis of TRAF4 and IL‐8 expression. D) Relative mRNA expression of *IL‐8* in HCT116, MDA‐MB‐231 and U‐87 MG cells overexpressing EV or NLS‐TRAF4. E) ELISA of IL‐8 in supernatants of HCT116, MDA‐MB‐231 and U‐87 MG cells overexpressing EV or NLS‐TRAF4. F) Relative mRNA expression of *IL‐8* in HCT116, MDA‐MB‐231 and U‐87 MG cells overexpressing EV, TRAF4 or TRAF4‐P12A. G) ELISA of IL‐8 in supernatants of HCT116, MDA‐MB‐231 and U‐87 MG cells overexpressing EV, TRAF4 or TRAF4‐P12A. H) The number of tumorspheres overexpressing EV, NLS‐TRAF4 or NLS‐TRAF4 + anti‐IL‐8(500 ng/mL). I) The number of tumorspheres overexpressing EV, NLS‐TRAF4 or NLS‐TRAF4 + CXCR1/2i (Reparixin, 100 nM) J) The number of tumorspheres overexpressing EV, TRAF4, TRAF4‐P12A or TRAF4‐P12A + rhIL‐8 (Recombinant human IL‐8 protein, 200 ng/mL). K) In vitro limiting dilution assay was performed in HCT116, MDA‐MB‐231 and U‐87 MG cells overexpressing EV, NLS‐TRAF4 or NLS‐TRAF4 + anti‐IL‐8(500 ng/mL). L) In vitro limiting dilution assay was performed in HCT116, MDA‐MB‐231 and U‐87 MG cells overexpressing EV, TRAF4, TRAF4‐P12A or TRAF4‐P12A + rhIL‐8 (Recombinant human IL‐8 protein, 200 ng/mL). M) Flow cytometry analysis of CD44^+^ cell ratio in different groups of HCT116, MDA‐MB‐231 and U‐87 MG cells. N) The transwell invasion assay was performed to analyze cell invasion of HCT116, MDA‐MB‐231 and U‐87 MG cells overexpressing EV, NLS‐TRAF4 or NLS‐TRAF4 + anti‐IL‐8(500 ng/mL). O) The transwell invasion assay was performed to analyze cell invasion of HCT116, MDA‐MB‐231 and U‐87 MG cells overexpressing EV, TRAF4, TRAF4‐P12A or TRAF4‐P12A + rhIL‐8 (Recombinant human IL‐8 protein, 200 ng/mL). Each experiment was performed at least three times. Statistical significance between two groups was determined with two tailed Student's *t*‐test. Statistical significance among groups was determined by one‐way ANOVA test. Data are presented as mean ± SD. ^*^ *p* < 0.05, ^**^ *p* < 0.01, ^***^ *p* < 0.001, n.s indicates non‐significant.

To investigate how nuclear TRAF4 promotes tumor stemness and dormancy, we conducted RNA sequencing on U87‐MG cells overexpressing NLS‐TRAF4. Our findings revealed that *IL‐8* was one of the most significantly upregulated genes, which is known to transform tumor cells into a mesenchymal phenotype (**Figure** **4**A). Based on this result, we hypothesized that *IL‐8* was the principal downstream gene of nuclear TRAF4 that mediates its role in tumor stemness and dormancy. Our analysis of the TCGA database showed a strong positive correlation between *TRAF4* and *IL‐8* gene expression in breast cancer and glioma (Figure 4B; Figure S4A, Supporting Information). Further validation supported our hypothesis, as evidenced by the positive association between TRAF4 and IL‐8 protein expression in breast cancer tissues (Figure 4C; Table S1 and Figure S4B, Supporting Information). Subsequently, we analyzed whether the transcriptomic changes were attributed to nuclear TRAF4. We verified that TRAF4 regulated IL‐8 at both RNA and secretory levels after overexpression of NLS‐TRAF4 in tumor cells (Figure 4D,E). We also observed that inhibiting TRAF4 nuclear accumulation suppressed the RNA and secretory levels of IL‐8 that TRAF4 increased in HCT116, MDA‐MB‐231, and U‐87 MG cells (Figure 4F,G). Interestingly, both the lL‐8 neutralizing antibody and the CXCR1/2 inhibitor could hinder tumor sphere formation induced by TRAF4 nuclear accumulation (Figure 4H,I; Figure S4C,D, Supporting Information). Moreover, reducing TRAF4 nuclear translocation diminished the tumor sphere formation induced by TRAF4, which was restored by recombinant IL‐8 (Figure 4J; Figure S3E, Supporting Information). Furthermore, in vitro limiting dilution experiments showed consistent phenomena (Figure 4K,L). We also discovered that TRAF4 nuclear accumulation increased the CD44^+^ cell population, while CXCR1/2 inhibitors decreased the population that was increased by NLS‐TRAF4 (Figure 4M; Figure S3F, Supporting Information). The IL‐8 neutralizing antibody effectively inhibited tumor cell invasion induced by TRAF4 nuclear accumulation (Figure 4N). Similarly, reducing TRAF4 nuclear translocation suppressed tumor cell invasion that was elevated by TRAF4, which was again restored by recombinant IL‐8 (Figure 4O). These data reveal that TRAF4 nuclear accumulation induces IL‐8 transcription and autocrine to maintain tumor stemness and dormancy.

### 2.5 Nuclear TRAF4 Interacts with C‐Jun to Facilitate IL‐8 Transcription

**Figure 5:**
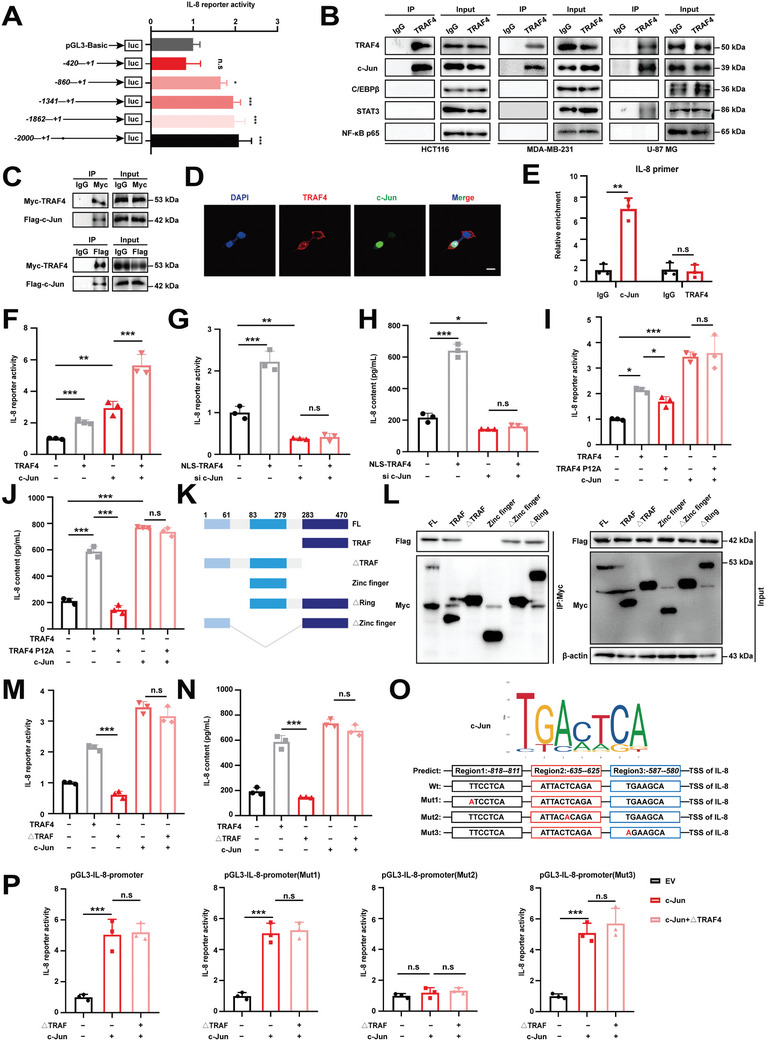
Nuclear TRAF4 Interacts with c‐Jun to Facilitate IL‐8 Transcription. A) Schematic representation of the different luciferase reporter constructs of the 5ʹ deletions IL‐8 promoter. The different 5ʹ deletions of the IL‐8 promoter luciferase reporter constructs were transfected into HEK‐293T cells stably expressing TRAF4 and luciferase activity was measured as described. B) Identification of transcription factors interacting with TRAF4 by Co‐IP assay. C) Co‐IP assays were conducted to investigate the interaction between exogenous TRAF4 and c‐Jun in HEK‐293T cells. D) Immunofluorescence assays were conducted to investigate the interaction between exogenous TRAF4 and c‐Jun in HEK‐293T cells. Scale bar, 150 µm. E) Chromatin from HCT116 cells transient expressing c‐Jun or TRAF4 was analyzed for recruitment of IL‐8 by ChIP‐qPCR. F) Analysis of IL‐8 promoter in response to TRAF4 or c‐Jun overexpression and luciferase activity was measured as described. G) Analysis of IL‐8 promoter in response to NLS‐TRAF4 overexpression or c‐Jun knockdown and luciferase activity was measured as described. H) ELISA of IL‐8 in supernatants of HCT116 cells overexpressing NLS‐TRAF4 or si c‐Jun. I) Analysis of IL‐8 promoter in response to TRAF4, TRAF4‐P12A or c‐Jun overexpression and luciferase activity was measured as described. J) ELISA of IL‐8 in supernatants of HCT116 cells overexpressing TRAF4, TRAF4‐P12A or c‐Jun. K) Schematic diagram illustrating the structural domains of TRAF4 and its truncated mutants. L) Co‐IP assays were conducted to investigate the binding regions of TRAF4 and c‐Jun. M) Analysis of IL‐8 promoter in response to TRAF4, △TRAF4 or c‐Jun overexpression and luciferase activity was measured as described. N) ELISA of IL‐8 in supernatants of HCT116 cells overexpressing TRAF4, △TRAF4 or c‐Jun. O) Sequence logo of c‐Jun reverse complement (top). Schematic representation of the different luciferase reporter constructs of the IL‐8 promoter mutants (bottom). P) Analysis of IL‐8 promoter specific mutants in response to △TRAF4 and c‐Jun overexpression. The specific mutants of the IL‐8 promoter luciferase reporter constructs were transfected into HEK‐293T cells and luciferase activity was measured. Each experiment was performed at least three times. Statistical significance between two groups was determined with two tailed Student's *t*‐test. Statistical significance among groups was determined by one‐way ANOVA test. Data are presented as mean ± SD. ^*^ *p* < 0.05, ^**^ *p* < 0.01, ^***^ *p* < 0.001, n.s indicates non‐significant.

To clarify how nuclear TRAF4 enhanced *IL‐8* expression, we tested whether TRAF4 promoted the transcriptional activity of IL‐8. Luciferase reporter assays showed that it did (**Figure** **5**A). Binding site deletion analysis of the IL‐8 promoter identified that the region between −860 and −420 was primarily responsible for the increased promoter activity induced by TRAF4. Since TRAF4 was not a transcriptional factor, we studied whether it interacted with any known transcriptional factors involved in regulating IL‐8 expression, including c‐Jun, C/EBPβ, STAT3, and NF‐κB.^[^ ^41^, ^42^, ^43^, ^44^ ^]^ Our results indicated that only c‐Jun could interact with TRAF4 in HCT116, MDA‐MB‐231, and U‐87 MG cells (Figure 5B). Furthermore, exogenous expression of TRAF4 and c‐Jun in HEK‐293T cells also confirmed their interaction, and we observed a positive correlation between nuclear TRAF4 expression and c‐Jun expression in BRCA tissues (Figure 5C,D; Figure S5A, Supporting Information). Chromatin immunoprecipitation (ChIP) results showed that c‐Jun, rather than TRAF4, was exclusively recruited to the IL‐8 promoter within the −860 to −420 region (Figure 5E). However, TRAF4 enhanced IL‐8 transcriptional activity that was already increased by c‐Jun, indicating that nuclear TRAF4 functioned as a co‐activator of c‐Jun (Figure 5F). We further found that silencing c‐Jun with siRNA eliminated the increased IL‐8 transcription and secretion resulting from TRAF4 nuclear accumulation (Figure 5G,H; Figure S5B, Supporting Information). Reducing TRAF4's nuclear translocation suppressed IL‐8 transcription and secretion elevated by TRAF4, which could be rescued by overexpressing c‐Jun (Figure 5I,J). We also found that the TRAF domain of TRAF4 was essential for its interaction with c‐Jun (Figure 5K,L). When the TRAF domain was lost, TRAF4 was unable to enhance IL‐8 transcription and secretion or further promote c‐Jun‐induced IL‐8 transcription and secretion (Figure 5M,N). Additionally, previous studies suggested that c‐Jun could upregulate several transcription factors that regulate tumor stemness and dormancy, particularly during the epithelial mesenchymal transition, such as SOX9, SOX2,^[^ ^45^ ^]^ Snail and Slug.^[^ ^46^ ^]^ Thus, we performed a ChIP assay to determine if nuclear TRAF4 increases c‐Jun's transcriptional activity on these downstream genes. The results showed that c‐Jun increased the transcription of SOX9, SOX2, Snail, and Slug, and TRAF4 further elevated their transcription levels induced by c‐Jun (Figure S5C, Supporting Information). Finally, the online tool Jaspar was used to predict 3 potential c‐Jun binding regions in the −860 to −420 region of the IL‐8 promoter (Figure 5O). Luciferase reporter assays for site‐directed mutagenesis showed that c‐Jun predominantly bound to region 2 rather than regions 1 or 3 (Figure 5P). These results demonstrated that both the TRAF domain of TRAF4 and region 2 of the IL‐8 promoter are essential for the transcriptional activation of IL‐8 by c‐Jun.

### 2.6 NGF Activated AKT to Phosphorylate TRAF4 at the S242 Site to Facilitate its Nuclear Accumulation

**Figure 6:**
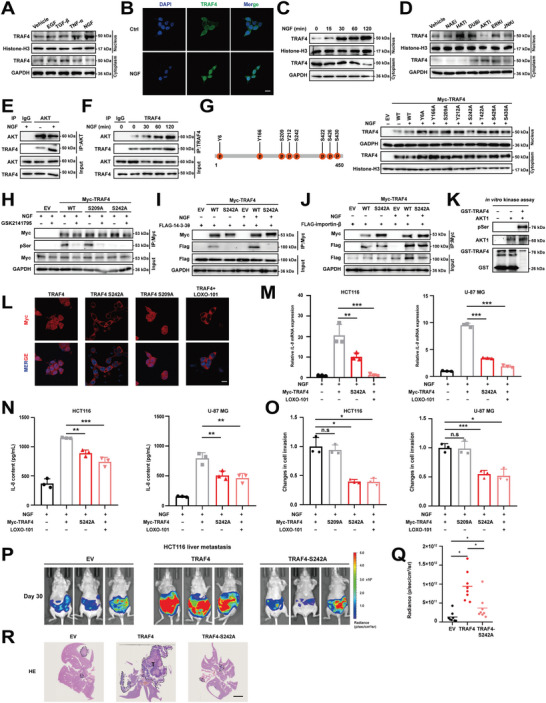
NGF activated AKT to phosphorylate TRAF4 at the S242 site to facilitate its nuclear accumulation. A) Expression of nuclear TRAF4 in HCT116 cells after administration of different cytokine stimuli detected by immunoblotting. B) NGF treatment promotes TRAF4 nuclear translocation. HCT116 cells were treated with or without NGF for 3 h and TRAF4 subcellular localization was examined via IFA assay. Scale bar, 150 µm. C) Time‐dependent analysis of nuclear TRAF4 by immunoblotting after NGF treatment in HCT116 cells. D) Expression of nuclear TRAF4 in HCT116 cells after administration of protein post‐translational modification inhibitors detected by immunoblotting. E) AKT interacts with TRAF4. HCT116 cells treated with NGF for 3 h were subjected to AKT immunoprecipitation. F) NGF treatment promotes TRAF4–AKT interaction. HCT116 cells were treated with NGF for indicated time points followed by TRAF4 Co‐IP assay. G) PhosphoSitePlus analysis (https://www.phosphosite.org) to predict key residues of TRAF4 phosphorylated by AKT. Schematic representation of the sites where TRAF4 may be phosphorylated by AKT (left). Expression of nuclear TRAF4 in HCT116 cells after transfer of TRAF4 plasmid with inactivating mutations at the phosphorylation site detected by immunoblotting. H) AKT inhibition attenuates NGF‐induced TRAF4 phosphorylation at Ser242. HCT116 cells were transiently transfected with indicated constructs for 24 h. After that, cells were pre‐treated with or without AKT inhibitor GSK2141795 for 6 h followed by NGF treatment for another 60 min. Cells were then subjected to Myc immunoprecipitation assay and analyzed via immunoblotting using indicated antibodies. I,J) HCT116 cells were co‐transfected with FLAG‐14‐3‐3θ and Myc‐TRAF4 for 24 h before processing for nuclear Co‐IP assay. HCT116 cells were co‐transfected with FLAG‐importin‐β and Myc‐TRAF4 for 24 h before processing for nuclear Co‐IP assay. K) AKT phosphorylates TRAF4 in vitro. Purified AKT kinase was incubated with recombinant GST or GST‐TRAF4 for 60 min before being subjected to immunoblotting analysis via indicated antibodies. L) pTRAF4^S242^ enhances the nuclear localization of TRAF4. HCT116 cells were transfected with indicated plasmids for 24 h before processing for IFA assay. LOXO‐101, a selective inhibitor of the tropomyosin‐related kinase (TRK) family receptors. Bar, 150 µm. M) Relative mRNA expression of *IL‐8* in HCT116 cells overexpressing EV, TRAF4 or TRAF4 S242A treated with NGF stimulation or LOXO‐101. N) ELISA of IL‐8 in supernatants of HCT116 cells overexpressing EV, TRAF4 or TRAF4 S242A treated with NGF stimulation or LOXO‐101. O) The transwell invasion assay was performed to analyze cell invasion of HCT116 overexpressing EV, TRAF4 or TRAF4 S242A treated with NGF stimulation or LOXO‐101. P,Q) A total of 2 × 10^6^ HCT116 cells overexpressing EV, TRAF4 or TRAF4 S242A were injected into the spleen to establish the colorectal cancer liver metastasis model. After 30 days, Bioluminescence was detected (P). Quantification of Bioluminescence (Q) is shown. R)Representative images of H&E staining from these groups of mice. Scale bar, 10 µm. Statistical significance among groups was determined by one‐way ANOVA test. Data are presented as mean ± SD. ^*^ *p* < 0.05, ^**^ *p* < 0.01, ^***^ *p* < 0.001, n.s indicates non‐significant.

To identify the upstream signaling that triggers the nuclear translocation of TRAF4, we stimulated tumor cells using several cytokines found in the tumor microenvironment (**Figure** **6**A). These four cytokines were selected because they are known to induce epithelial‐mesenchymal transition and activate TRAF4‐related signaling.^[^ ^36^, ^38^, ^47^, ^48^ ^]^ Our findings indicated that NGF signaling led to the accumulation of TRAF4 in the nucleus. Immunofluorescence analysis exhibited a significant increase in nuclear accumulation of TRAF4 in both HCT116 and U‐87 MG cells (Figure 6B; Figure S6A, Supporting Information). The protein levels of nuclear TRAF4 were notably increased 15 min after NGF treatment, remaining elevated up to 120 min (Figure 6C; Figure S6B, Supporting Information). Given that NGF signaling can influence multiple cellular pathways, we employed various inhibitors to identify the direct downstream pathway responsible for NGF‐induced TRAF4 nuclear accumulation. Our results demonstrated that the AKT inhibitor could effectively suppress TRAF4 nuclear translocation in tumor cells (Figure 6D; Figure S6C, Supporting Information). Thus, we hypothesized that AKT directly binds to TRAF4 to facilitate its nuclear translocation. An immunoprecipitation assay confirmed that the interaction between TRAF4 and AKT was significantly increased after NGF treatment (Figure 6E; Figure S6D, Supporting Information), and this interaction further intensified over time (Figure 6F; Figure S6E, Supporting Information). Since AKT is a kinase, we wondered whether AKT directly phosphorylated TRAF4. The interactions between TRAF4 and c‐Jun or AKT were confirmed in RKO, LM2‐4175, and LN229 cells with high nuclear TRAF4 expression, showing interactions at endogenous levels (Figure S6F, Supporting Information). Using PhosphoSite for prediction, we selected the seven most likely phosphorylation sites on TRAF4 linked to NGF/AKT signaling for point mutation analysis. NGF increased the nuclear translocation of wt‐TRAF4 and other mutants except for TRAF4‐S242A (Figure 6G). We observed elevated TRAF4 phosphorylation in wt‐TRAF4 and TRAF4‐S209A‐expressing cells, which was blocked by further treatment with an AKT inhibitor (Figure 6H). However, the TRAF4‐S242A mutation completely suppressed NGF‐induced TRAF4 phosphorylation regardless of AKT inhibitor treatment, indicating that AKT phosphorylated TRAF4 at the S242 site. NGF‐induced phosphorylation of TRAF4 at the S242 site enhanced TRAF4's interaction with 14‐3‐θ, facilitating its nuclear translocation instead of importin‐β (Figure 6I,J). An in vitro kinase also confirmed that active AKT was able to phosphorylate TRAF4 (Figure 6K). As NGF must bind to its receptor to activate downstream AKT signaling, we tested LOXO‐101,^[^ ^49^ ^]^ a selective inhibitor of the NGFR family, to inhibit TRAF4 nuclear accumulation. Compared with TRAF4‐expressing cells, TRAF4‐S242A impeded its nuclear translocation (Figure 6L; Figure S6G, Supporting Information), leading to decreased IL‐8 transcription (Figure 6M) and reduced secretion (Figure 6N), and impaired cell invasion (Figure 6O). LOXO‐101 produced effects similar to those of the S242 mutation. In the liver metastasis model of HCT116 cells, TRAF4 promoted liver metastasis, whereas TRAF4‐S242 inhibited it (Figure 6P–R). These data indicated that NGF‐activated AKT to phosphorylate TRAF4 at the S242 site, facilitating its nuclear accumulation and promoting metastasis.

### 2.7 Pharmacological Inhibition of Nuclear TRAF4 Suppresses Tumorigenesis and Metastasis

**Figure 7:**
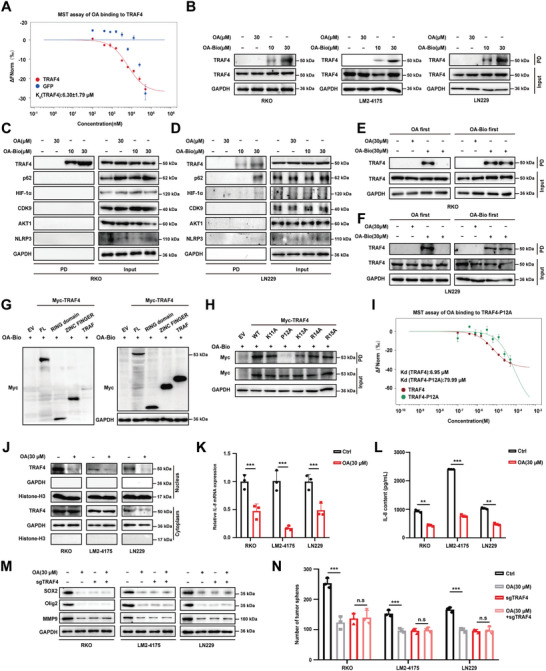
Pharmacologic targeting of nuclearTRAF4 inhibits tumorigenesis and metastasis. A) MST assay for the affinity between OA and purified GFP‐TRAF4 protein. B) Cell lysates of HCT116, MDA‐MB‐231 and U‐87 MG cells were incubated with OA (30 µM) and different concentrations of OA‐Bio (10 µM, 30 µM), which were then pulled down using streptavidin beads. C,D) Comparison of some of the other reported targets of OA. Cell lysates of HCT116, MDA‐MB‐231 and U‐87 MG cells were incubated with OA (30 µM) and different concentrations of OA‐Bio (10 µM, 30 µM), which were then pulled down using streptavidin beads. E,F) Cell lysates of RKO and LN229 were incubated with different concentrations of free OA (30 µM) for 3 h before or after incubated with OA‐Bio (30 µM) for 3 h, and then pulled down using streptavidin beads. G) HEK‐293T cell lysates overexpressing full length or various mutants of TRAF4 were incubated with OA‐Bio and then pulled down using streptavidin beads. H) TRAF4‐WT, TRAF4‐K11A, TRAF4‐P12A, TRAF4‐K13A, TRAF4‐R14A and TRAF4‐R15A with Myc were transfected in HEK‐293T cells, which were co‐incubated with 30 µM OA‐Bio and their cell lysates, respectively, and the binding sites between OA and TRAF proteins were detected by Pull‐Down assay and immunoblotting assay. I) MST assay for the affinity between OA and purified GFP‐TRAF4/GFP‐TRAF4‐P12A protein. J) RKO, LN229 and LM2‐4175 cells were treated with 30 µM OA for 48 h. The expression of nuclear TRAF4 was detected by nuclear plasma separation assay and immunoblotting. K) Relative mRNA expression of *IL‐8* in RKO, LN229 and LM2‐4175 cells treated with 30 µM OA for 48 h. L) ELISA of IL‐8 in supernatants of RKO, LN229 and LM2‐4175 cells treated with 30 µM OA for 48 h. M) TRAF4‐silenced RKO, LN229 and LM2‐4175 cells were treated with 30 µM OA. Immunoblotting analysis of the expression of TRAF4. N)The number of tumorspheres in TRAF4‐silenced RKO, LN229 and LM2‐4175 cells were treated with 30 µM OA. Each experiment was performed at least three times. Statistical significance between two groups was determined with two tailed Student's *t*‐test. Statistical significance among groups was determined by one‐way ANOVA test. Data are presented as mean ± SD. ^*^ *p* < 0.05, ^**^ *p* < 0.01, ^***^ *p* < 0.001, n.s indicates non‐significant.

At the end of this study, we aimed to find a potential inhibitor of nuclear TRAF4 by testing the binding affinity between TRAF4 and several natural products known for their anti‐metastatic properties.^[^ ^50^, ^51^, ^52^, ^53^, ^54^, ^55^, ^56^, ^57^, ^58^, ^59^ ^]^ The results showed that Oroxylin A (OA) had the smallest equilibrium dissociation constant (Kd) of ≈6.30 µM, indicating that it could potentially be a specific inhibitor of TRAF4 (**Figure** **7**A; Figure S7A, Supporting Information). To confirm that OA specifically targets TRAF4, we synthesized biotinylated‐OA (OA‐Bio) (Figure S7B–D, Supporting Information) and demonstrated its ability to pull down purified TRAF4 protein in vitro (Figure S7E, Supporting Information). We found that TRAF4 was effectively pulled down by OA‐Bio in several cell types with high levels of nuclear TRAF4 in a concentration‐dependent manner (Figure 7B). Exogenous expression of TRAF4 was also successfully pulled down by OA‐Bio (Figure S7F, Supporting Information). Notably, the previously reported target of OA, except p62 in one cell type, could not be pulled down by OA‐Bio (Figure 7C,D). Additionally, among the TRAF family, only TRAF4 was pulled down by OA‐Bio (Figure S7G, Supporting Information). Further treatment of OA reversed the pull‐down of TRAF4 by OA‐Bio. However, when lysates were preincubated with OA‐Bio, subsequent treatment of OA could not prevent their interaction, indicating a firm binding (Figure 7E,F). We also explored the binding domains of TRAF4 that interacted with OA and found that only the RING and ZINC FINGER domains, where the two nuclear localization sequences are located, could interact with OA (Figure 7G). To identify the cysteine residue responsible for binding, we utilized the mutants of the nuclear localization sequence of TRAF4. The results showed that P12A abolished TRAF4 binding to OA, suggesting that proline at position 12 was the critical binding residue and is also essential for TRAF4 nuclear translocation (Figure 7H). The Microscale Thermophoresis (MST) assay confirmed that mutation of P12 resulted in a significantly decreased Kd (Figure 7I). We further validated the binding affinity between OA and TRAF4 using the Isothermal Titration Calorimetry (ITC) assay, which determined the dissociation constant Kd to be 1.1 µM. (Figure S7H, Supporting Information). We then tested the interaction of OA with other TRAF family members using the MST assay. The results showed that OA is specifically bound to TRAF4, with no binding observed for TRAF1, TRAF2, or TRAF6 (Figure S7I, Supporting Information), reinforcing the specificity of OA targeting TRAF4. We found that OA could inhibit TARF4 nuclear accumulation (Figure 7J) and consequently suppress the transcription and secretion of IL‐8 (Figure 7K,L). Furthermore, when TRAF4 was knocked out, OA could no longer inhibit the expression of SOX2, Olig2, and MMP9 (Figure 7M), nor did it impair tumor sphere formation (Figure 7N).

**Figure 8:**
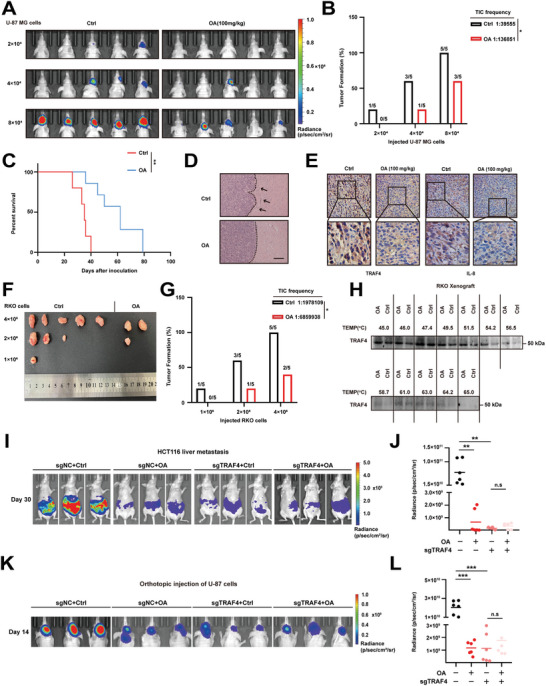
Pharmacological Inhibition of Nuclear TRAF4 Suppresses Tumorigenesis and Metastasis. A–C) In vivo bioluminescent images (A) of luciferase‐labeled U‐87 MG derived xenografts in the brains of mice treated with OA (100 mg k^−1^g) or the vehicle control after 10 days. Tumor‐inducing incidence B) and overall survival curves C) are shown. D) Representative images of H&E staining from the brains of mice treated with OA (100 mg k^−1^g) or the vehicle control. Scale bar, 100 µm. E) IHC of tumor tissues showing TRAF4 and IL‐8 expression. Scale bar, 100 µm. F,G) Representative images of tumors (F) and effects of the treatment with OA (100 mg k^−1^g) on tumor‐inducing incidence (G) in the RKO CDX mouse models (n = 5 mice per group). H) CETSA melt curve of TRAF4 for heat treatment of tumors in the RKO CDX mouse models in the absence and in the presence of OA. I) Control or TRAF4‐KO HCT116 cells were injected into the spleen to establish the liver metastasis of colon cancer. 1 day later, mice were treated with vehicle control and OA at the indicated time points. After 30 days, Bioluminescence was detected. J) Quantification of Bioluminescence is shown. I) Control or TRAF4‐KO U‐87 MG cells were orthotopically injected into brain. 1 day later, mice were treated with vehicle control and OA at the indicated time points. After 14 days, Bioluminescence was detected. J) Quantification of Bioluminescence is shown. Statistical significance among groups was determined by one‐way ANOVA test. Data are presented as mean ± SD. ^*^ *p* < 0.05, ^**^ *p* < 0.01, ^***^ *p* < 0.001, n.s indicates non‐significant compared to control group.

In vivo limiting dilution assays demonstrated that OA diminished the tumorigenic capability of U87‐MG cells in mice after orthotopic transplantation (**Figure** **8**A,B). Additionally, OA prolonged the survival of the mice (Figure 8C) and reduced the presence of dormant tumor cells at the invasive front (Figure 8D). Furthermore, OA decreased the protein levels of nuclear TRAF4 and IL‐8 (Figure 8E). Consistently, OA also inhibited the tumorigenesis of RKO cells in mice by subcutaneous inoculation (Figure 8F,G). In the CETSA assay, TRAF4 expression gradually disappeared as the temperature increased in RKO tumors (Figure 8H). Notably, a significant reduction in TRAF4 expression was observed at 54.2 °C due to the interaction between TRAF4 and OA. To determine whether the anti‐tumor effect of OA depended on TRAF4, TRAF4‐KO HCT116, and U‐87 MG cells were used to establish tumor models. Compared with the control group, both OA treatment and TRAF4 knockout could inhibit liver metastasis of colon cancer cells and reduce the tumorigenicity of glioma cells (Figure 8I–L). However, OA could not further prevent the metastasis and tumorigenesis in TRAF4‐KO cells, which indicated that OA inhibited tumor metastasis and initiation by targeting TRAF4. These results demonstrate that OA can potentially be used as a pharmacologic inhibitor of TRAF4, which can effectively hinder tumorigenesis and metastasis in tumor cells.

## 3 Discussion

The recurrence and metastasis of malignant tumors are the primary factors contributing to the failure of tumor therapies and cancer‐associated deaths, with metastatic dormancy playing an important role.^[^ ^11^ ^]^ Tumor dormancy refers to a state of tumor that occurs in various kinds of tumors,^[^ ^60^, ^61^, ^62^ ^]^ characterized by either quiescence or a balance between cell division and apoptosis.^[^ ^11^, ^27^, ^63^ ^]^ Studies have discovered that there are many similar characteristics and regulatory pathways between stemness and tumor dormancy.^[^ ^61^, ^64^, ^65^ ^]^ Therefore, inhibition tumor cells from acquiring stemness may provide a new strategy for the prevention of tumor dormancy and removal of minimal residual lesions. TRAF4 is the only member in the TRAF protein family with a nuclear localization sequence^[^ ^40^ ^]^ and nuclear TRAF4 was specifically expressed in tumor tissues.^[^ ^34^, ^36^, ^37^ ^]^ However, the function and mechanism of nuclear TRAF4 in tumor progression remain unclear. In this study, we found that nuclear TRAF4 expression was higher in multiple human cancer tissues and cell lines compared with their matched normal tissues and cells. Nuclear TRAF4 served as an independent indicator of tumor aggressiveness and differentiation, acting as a poor prognostic factor. Key molecules such as NR2F1,^[^ ^64^ ^]^ DEC2,^[^ ^66^ ^]^ p27,^[^ ^67^ ^]^ and SOX9^[^ ^68^ ^]^ play critical roles in dormancy induction and maintenance, and SOX2 and OLIG2 are key regulators in stemness characteristics.^[^ ^69^ ^]^ Nuclear TRAF4 could not only enhance these biomarkers and facilitate the acquisition of a mesenchymal phenotype but also induce metastatic dormancy.^[^ ^34^ ^]^ To thoroughly investigate the unique function of nuclear TRAF4,^[^ ^36^ ^]^ we expressed both nuclear and cytoplasmic TRAF4 in TRAF4‐KO cells. However, our findings still could not completely exclude the influence of cytoplasmic TRAF4 on tumor development, because studies have shown that cytoplasmic TRAF4 could promote metastasis by activating TGF‐β, TrkA, and other cytoplasmic signals.^[^ ^36^, ^37^ ^]^ Indeed, TRAF4 nuclear accumulation should not be viewed as a separate molecular event. It is often associated with TRAF4 gene amplification and high expression, followed by the nuclear translocation of TRAF4 from the cytoplasm. As tumors develop, TRAF4 in the cytoplasm and nucleus may undergo dynamic changes. During cell growth and invasion, cytoplasmic TRAF4 levels increase, which enhances cell proliferation and motility. When cells reach a metastatic lesion, they receive signals from IL‐8 produced by stromal cells in that lesion, which promotes the entry of TRAF4 into the nucleus and initiates the process of tumor dormancy. If the implanted tumor cells need to proliferate, TRAF4 will return to the cytoplasm and will not re‐enter the nucleus until it receives further stimulation from the microenvironment. Thus, our study demonstrated a new mechanism by which tumors maintain a dormant state, supporting the idea that stemness and EMT can contribute to this dormancy in tumors.

To investigate the function of nuclear TRAF4 and its underlying mechanisms, we focused on c‐Jun, a transcription factor that has been reported to be positively correlated with TRAF4. A previous study showed that cytoplasmic TRAF4 induced degradation‐independent K63‐linked ubiquitination to activate JNK1/2 kinase.^[^ ^70^ ^]^ Nuclear TRAF4, on the other hand, interacts directly with c‐Jun through its TRAF domain, functioning as a transcriptional co‐activator. The TRAF domain of TRAF4 has also been reported to bind with other partner proteins.^[^ ^34^ ^]^ While both cytoplasmic and nuclear TRAF4 could activate JNK signaling, the mechanisms by which they do so are entirely different. Furthermore, TRAF4 accelerated c‐Jun‐meditated transcription and secretion of IL‐8, which was responsible for nuclear TRAF4‐induced stemness and metastasis. Although TRAF4 is a key regulator in inflammatory signaling^[^ ^71^, ^72^, ^73^ ^]^ and could produce various interleukins such as IL‐33 and IL‐25,^[^ ^74^, ^75^ ^]^ its specific role in promoting IL‐8 secretion was not understood. IL‐8 is secreted by various cancers and can stimulate cancer cell metastasis in an autocrine fashion.^[^ ^76^, ^77^, ^78^, ^79^ ^]^ Our study found that both the IL‐8 neutralizing antibody and its receptor CXCR1/2 inhibitor could eliminate nuclear TRAF4‐promoted stemness and invasion, highlighting that targeting IL‐8/CXCR1/2 was a promising strategy for metastatic cancer.

In our study, we aimed to identify the triggers of TRAF4 nuclear translocation and discovered that NGF plays a key role. NGF is a neurotrophic factor involved in the regulation of neuronal functions through the binding of NGFR.^[^ ^80^ ^]^ Evidence has shown that NGF can also be synthesized and secreted by stromal cells of the tumor environment, such as stellate cells.^[^ ^81^, ^82^ ^]^ We found that NGF induced TRAF4 phosphorylation at S242 by AKT, which promoted its interaction with 14‐3‐3θ to transport TRAF4 into the nucleus where it interacts with c‐Jun to regulate IL‐8 transcription. This process was similar to the nuclear translocation of TSPAN8 in breast cancer.^[^ ^83^ ^]^ In this study, our results showed that NGF/NGFR and AKT were upstream activators of TRAF4. This relationship may seem controversial as TRAF4 could promote NGFR and AKT activation in a ubiquitination‐dependent manner.^[^ ^36^, ^84^ ^]^ We believe that this dynamic reflects a positive feedback loop characterized by cross‐signaling in tumor cells. In this loop, the proteins interact and activate each other, fostering a vicious cycle often associated with malignant progression. Importantly, we noted that the above process of TRAF4 nuclear translocation could be inhibited by LOXO‐101, an NGFR inhibitor that has been approved for the treatment of metastatic solid tumors regardless of the site of tumor occurrence. It is a sideways way of proving that nuclear TRAF4‐promoting metastasis is a common phenomenon in pan‐tumors.

Since blocking TRAF4 phosphorylation only partially impaired TRAF4 nuclear accumulation and considering that LOXO‐101 treatment imposes a financial burden on patients, we decided to explore potential TRAF4 inhibitors derived from natural products. One candidate is OA, a flavonoid compound isolated from the root of *Scutellaria Baicalensis* that has been reported to possess antitumor activities in various tumors and has already been approved for clinical trials.^[^ ^85^, ^86^, ^87^ ^]^ Although previous studies suggested that OA could regulate multiple cellular pathways, its direct molecular target remained unknown. In our study, we found that OA could specially bind to TRAF4 and OA‐mediated inhibition of tumor sphere formation is dependent on TRAF4 expression. This indicated that TRAF4 was the main target of OA. Notably, our results showed that OA could interact with P12 of the nuclear localization sequence of TRAF4, which led to reduced TRAF4 nuclear translocation. This may occur because the hydroxyl group of OA coincidentally hydroxylates the proline, leading to the inactivation of the nuclear localization sequence, which is located in the RING domain. The interaction of OA with another nuclear localization sequence of the ZINC FINGER domain was not examined in this study. Most importantly, OA treatment significantly inhibited tumorgenesis and metastasis and provided survival benefits in tumor‐bearing mice, which supported the potential of OA for clinical application in the treatment of recurrent and metastatic tumors as a monotherapy or adjuvant therapy.

## 4 Experimental Section

### Antibodies

All antibodies used in this study were purchased from commercial companies. They were anti‐TRAF4 antibody (RRID: AB_2798802), anti‐GAPDH antibody (RRID:AB_2736879), anti‐β‐actin antibody (RRID:AB_2863784), anti‐Histone H3 antibody (RRID:AB_2770395), anti‐Myc antibody (RRID: AB_2734122), anti‐GST antibody (RRID:AB_2771923), anti‐Olig2 antibody (RRID:AB_2759654), anti‐SOX2 antibody (RRID: AB_10999918), anti‐MMP9 antibody (RRID:AB_2758590), anti‐GFP antibody (RRID:AB_11042881), anti‐AKT antibody (RRID:AB_2861912), anti‐pSer/Thr antibody (RRID:AB_330308), anti‐IL‐8 antibody (RRID:AB_2249110), anti‐STAT3 antibody (RRID:AB_2758451), anti‐ DDDDK‐tag (RRID: AB_2770401), anti‐C/EBPβ antibody (RRID:AB_2757357), anti‐NF‐κB p65/RelA antibody (RRID:AB_2758145), anti‐p62 antibody (RRID:AB_2862742), anti‐HIF‐1α antibody (RRID:AB_2622225), anti‐CDK9 antibody (RRID:AB_2732887), anti‐NLRP3 antibody (RRID:AB_2766412), anti‐c‐Jun antibody (RRID:AB_2757059), and anti‐NR2F1 antibody (RRID:AB_2770655), anti‐SOX9 antibody (RRID:AB_2862748), anti‐P27 antibody (RRID:AB_2766167), and anti‐DEC2 antibody (RRID:AB_2758097).

### Cells, Cell Culture, and Tissues

Human colon cancer cell lines (HCT116, RRID: CVCL_0291; RKO, RRID: CVCL_0504), human glioma cell lines (LN229, RRID: CVCL_A8V5; U‐87 MG, RRID: CVCL_0022) and human breast cancer cell lines (MDA‐MB‐231, RRID: CVCL_0062) and HEK‐293T (RRID: CVCL_0063) cell line were purchased from the Cell Bank of the Chinese Academy of Sciences (Shanghai, China). Human breast cancer cell lines (LM2‐4175 derived from MDA‐MB‐231) were a gift from professor Guohong Hu's lab at the Shanghai Institute of Nutrition and Health, Chinese Academy of Sciences. All cells were cultured in a medium supplemented with 10% FBS (Wisent, Nanjing, China). All cells were incubated at 5% CO_2_ at 37 °C. All cell lines were authenticated via STR profiling and were tested negative for mycoplasma contamination.

BRCA surgical specimens and adjacent tissues were collected with the approval of the Institutional Ethics Review Boards of the First Affiliated Hospital of Nanjing Medical University in accordance with the guidelines of the International Ethical Guidelines for Biomedical Research Involving Human Subjects (CIOMS). Written informed consent was obtained from all patients (Ethics No.2021‐SR‐308). Commercial GBM tissue microarray containing a total of 46 case samples (39 gliomas and 7 normal brain tissues) with follow‐up data was from Alena Biotechnology. Commercial BRCA tissue microarray containing a total of 49 case samples (42 BRCA tissues and 7 normal mammary tissues) with follow‐up data from Weiao Biotechnology. Commercial COAD tissue microarray containing a total of 148 case samples (74 COAD tissues and 74 normal adjacent tissues) with follow‐up data from Aifang Biotechnology.

### Animals and Treatments

Female, 5‐week‐old BALB/c nude mice were purchased from Ziyuan Laboratory Animal Technology. Animals were housed in specific pathogen‐free conditions and fed standard mouse chow. All animal experiments were approved by the Institutional Animal Care and Use Committee at China Pharmaceutical University (Nanjing, China). All procedures for animal experiments were performed in accordance with the Guide for the Care and Use of Laboratory Animals and according to the institutional ethical guidelines for animal experiments.

### Trans‐Splenic Liver Metastasis Models

For trans‐splenic liver metastasis models, nude mice were randomly grouped (n ≥ 6 in each group), and their spleens were completely exposed via a ventral midline incision after being anesthetized and routinely disinfected. Luc‐labeled HCT116 or RKO cells (2 × 10^6^ per mouse) in 100 µL PBS were slowly injected into the spleen subcapsule. A total of four weeks later, mice were anesthetized with isoflurane and intraperitoneally injected with 150 mg kg^−1^ D‐luciferin potassium salt (Yeasen, 40901ES01), and tumors were imaged by IVIS spectrum apparatus (Perkin‐Elmer). For the EdU labeling assay, nude mice were injected with 2 × 10^6^ cells injected into the spleen subcapsule and were intraperitoneally injected with 100 ng/mouse EdU (Beyotime, ST067) 24 h before liver harvest.

### In Vivo Limiting Dilution Assay

For in vivo limiting dilution assay (LDA), U‐87 MG orthotopic xenograft tumor, 2 × 10^4^, 4 × 10^4^, and 8 × 10^4^ U‐87 MG‐Luc cells were intracranially injected into BALB/c nude mice, respectively. A total of 10 days later, mice were anesthetized with isoflurane and intraperitoneally injected with 150 mg kg^−1^ D‐luciferin potassium salt (Yeasen, 40901ES01), and tumors were imaged by IVIS spectrum apparatus (Perkin‐Elmer, USA). For animal survival analysis, mice were sustained until showed neurologic symptoms because of tumor burden or until the end of the experiment.

### Plasmid Construction, Transfection, and Lentiviral Infection

Cells were seeded in 6‐well plates. After confluence reached 50%, siRNA or plasmids were transfected by using Lipofectamine RNAiMAX or Lipofectamine 2000 (Thermo Fisher Scientific, 11668027). c‐JUN siRNAs were synthesized by Tuoran Biotechnologies.

c‐JUN, TRAF4, TRAF4 Y6A/Y166A/S209A/Y212A/S242A/T422A/S426A/S430A, TRAF4‐NLS, TRAF4‐K11A/P12A/K13A/R14A/R15A, TRAF4‐ΔRing, Ring, ΔZinc finger, ΔTRAF, GFP‐TRAF4, TRAF4 short hairpin RNA (shRNA) or TRAF4 overexpression lentivirus plasmids and IL‐8 promoter truncated plasmids were generated in the lab. The primer sequences are listed in Table S2 (Supporting Information). A lentiviral packaging kit (Yeasen, 41102ES) was used to transfect lentiviral plasmids into HEK‐293T cells. Then viral media produced by HEK‐293T was collected after 48 h and target cells were infected.

### Immunoblotting, Immunofluorescence Staining, Immunohistochemistry, and Hematoxylin and Eosin (H&E) Staining

Immunoblotting was carried out following previous methods.^[^ ^88^ ^]^ Briefly, cells or tissues were lysed in RIPA buffer (Thermo Fisher Scientific, 89901) supplemented with protease inhibitors (MCE, HY‐17541). Then proteins were separated by SDS‐PAGE. Membranes were blocked by 3% skim milk and then incubated with indicated antibodies.

For immunofluorescence (IF) staining assay, cells or tumor spheroids were fixed with 4% paraformaldehyde then permeabilized in Triton X‐100 (Beyotime, P0096) for 20 min and blocked with 5% BSA. Then incubated with indicated antibodies overnight. The next day, cells or spheroids were washed three times and incubated secondary antibodies (Alexa flour‐488, Alexa flour‐546) for 1 h at room temperature. DAPI (Beyotime, P0131,) was used to dye the cell nucleus. All samples were photographed by Fluoview FV 1000 confocal laser scanning microscope (Olympus, Japan).

For immunohistochemistry (IHC) staining, the tissue microarrays were baked at 60 °C for 120 min, then deparaffinized and hydrated with xylene and ethanol. The microarrays were then microwaved in citrate buffer to remove antigens. Endogenous peroxidase was blocked with a total of 3% H_2_O_2_ and then the microarrays were blocked with BSA. The microarrays were then incubated with the indicated primary antibodies overnight. The next day, the microarrays were washed three times with PBS and then incubated with the secondary antibody. Samples were stained with diaminobenzidine and hematoxylin. All tissue samples were scanned with Nanozoomer 2.0 (Hamamatsu, Japan).

For histological assays, mouse tumor tissues were fixed with 4% PFA and submitted to Wuhan ServiceBio Technology Co., Ltd for paraffin embedding. The embedded tissues were sectioned at 4 µm, deparaffinized, and subjected to hematoxylin and eosin (H&E) staining.

### 3D Tumor Spheroid Formation

A total of 4000 single tumor cells were seeded into 24‐well ultralow attachment plates (Corning, 3473) and incubated at 5% CO_2_, 37 °C in DMEM/F12 supplemented with 20 ng mL^−1^ EGF, 20 ng mL^−1^ bFGF, 2% B27, and 1% methylcellulose.

### In Vitro Extreme Limiting Dilution Assay

Tumor cells were seeded on 96‐well plates at 5, 10, 20, 50, 100, 200, and 500 cells per well. After incubating for 7 days, each well was examined for tumor spheroid formation. Stem cell frequency was calculated using extreme limiting dilution analysis (ELDA). (http://bioinf.wehi.edu.au/software/elda/).

### Nuclear and Cytoplasmic Protein Separation Experiment

Cells were harvested and resuspended in the hypotonic lysate (10 mM Tris‐HCl pH = 7.5, 3 mM MgCl_2_, 10 mM NaCl, 10% glycerol, 0.3% NP‐40, and protease inhibitor before use), mixed well, and lysed on ice for 60 min. The supernatant was centrifuged at 13 000 rpm for 20 min at 4 °C and the supernatant obtained from centrifugation was the cytoplasmic protein. The precipitates were washed with hypotonic lysate three times and then centrifuged to collect the nuclei. The nuclei were lysed by adding 2% SDS, and the nuclei were fully fragmented by ultrasonication. The supernatant was centrifuged at 13 000 rpm for 20 min at 4 °C, and the resultant supernatant was the nuclear protein. After that, the isolated parts were processed for western blotting analysis or Co‐IP assay as stated.

### Immunoprecipitation

Cells were lysed in 50 mmol L^−1^ HEPES, 150 mmol L^−1^ EDTA, 0.5% NP‐40, 1 mmol L^−1^ phenylmethylsulfonylfluoride, 1 × protease inhibitor at 4 °C for 1 h. Lysates were centrifuged at 13 000 rpm for 20 min at 4 °C. The supernatant was collected and incubated with the appropriate antibodies that were previously immobilized on Protein A/G Magnetic Beads (MCE, HY‐K0202) at 4 °C with rotation overnight. The beads were washed 4 times boiled with loading buffer and detected by immunoblotting.

### Luciferase Reporter Assay

The pGL3 plasmid and pRLTK plasmid were transfected into cells using Lipofectamine 2000 reagent. Then cells were assayed by using a Dual Luciferase Reporter Assay Kit (Vazyme, DL101‐01). Luciferase intensity was detected with a Luminoskan Ascent (Thermo Fisher Scientific, USA). The primer sequences of the different 5ʹ deletions of the IL‐8 promoter luciferase reporter plasmids and the mutant IL‐8 promoter luciferase reporter plasmids are listed in Table S2 (Supporting Information).

### Cellular Thermal Shift Assay

Cells with indicated treatment were collected with cold PBS supplemented with protease inhibitor then transferred into PCR tubes to be heat shocked at indicated temperature for 3 min and cooled down at room temperature for 3 min. Next, samples were repeated freezing and thawing cycle three times by using liquid nitrogen to lyse cells. After centrifuged, cell lysate was boiled with loading buffer and detected by immunoblotting assay.

### Microscale Thermophoresis Ligand‐Binding Assay and Drug Screening

HEK‐293T cells expressing GFP‐TRAF4 or GFP were lysed in RIPA buffer supplemented with protease inhibitors. Cell lysates were diluted in PBST and fluorescent signals of GFP proteins were detected by Monolith NT.115 (NanoTemper, Germany). Then GFP proteins and indicated ligands were mixed and loaded into NT.115 standard coated capillaries (NanoTemper, Germany). For drug screening, drugs were mixed with GFP‐TRAF4, respectively. The K_D_ value was calculated by fitting a standard binding curve.

### ELISA

Tumor cell culture supernatant (three sub‐wells per group) was collected, and then the Bicinchoninic Acid (BCA) Kit (Thermo Fisher Scientific,23227) was used to determine the total protein concentrations. The levels of interleukin‐8 (IL‐8) were then quantified using a commercially available ELISA kit (Abclonal, RK00011) according to the manufacturers' protocol.

### Chromatin Immunoprecipitation (ChIP) Assay

Cells were cross‐linked with 1% formaldehyde, quenched with 125 mM glycine, rinsed with PBS, sonicated with a Covaris M220 sonicator (Covaris, USA), and then centrifuged at 4 °C. DNA was fragmented to ≈250 bp. Protein A/G beads were pre‐incubated overnight at 4 °C with IgG or IL‐8 antibody. Finally, the immune complexes were eluted with elution buffer (1% SDS, 0.1 m NaHCO_3_, pH 8.4) for 10 min at room temperature. Reverse cross‐linking was performed in high‐salt buffer (0.2 M NaCl, 50 mM Tris, pH 6.5, 10 mM EDTA, 0.2 mg m^−1^; proteinase K [Beyotime, ST532]) at 65 °C overnight. Extracted and purified immunoprecipitated DNA was quantified by qRT‐PCR.

### Flow Cytometry Analyses

For flow cytometry, cell suspensions from tumor cells were directly labeled. Fluorescently labeled antibodies against the following surface proteins were used for cell staining: FITC‐CD44 (RRID: AB_465044). Then, cells were washed, and data were performed with a Becton Dickinson FACS Calibur flow cytometer using Cell‐Quest software (BD Biosciences). Data were analyzed with FlowJo v.10.

### GSEA

Genes were ranked by their association with U‐87 MG EV cells versus U‐87 MG NLS‐TRAF4 cells and using the signal‐to‐noise metric determined by GSEA according to the log_2_ fold change values.

### Invasion Assay

Transwell assays were performed using Millicell inserts (8.0 µm, Millipore) to evaluate cell invasion. Millicell inserts were precoated with 10 µg mL^−1^ fibronectin and Matrigel (1:6; Yeasen, 40183ES08) and then allowed to dry. Millicell inserts were placed into the wells of a 24‐well plate containing culture medium supplemented with 10% FBS. Cells (5 × 10^4^ cells/well) were starved overnight and were then seeded in the upper chambers without FBS culture medium. In total, 24 h later, the migrated cells were fixed with paraformaldehyde, stained with 0.1% crystal violet, and counted.

### Pull‐Down Assay

For the endogenous interaction assay, tumor cells were stimulated and lysed with NP‐40 lysis buffer with a complete protease inhibitor. The cell lysates were incubated overnight at 4 °C with the primary antibodies and Streptavidin Magnetic Beads. The proteins bound by the antibody were pulled down by Streptavidin Magnetic Beads and subjected to immunoblotting analysis. For the exogenous interaction assay, HEK‐293T cells were transfected with plasmids in 6‐well plates via Lipofectamine 2000 (Thermo Fisher Scientific, 11668500). After 24 h, cells were collected and lysed with NP‐40 lysis buffer with complete protease inhibitor. The proteins bound by the antibody were pulled down by Streptavidin Magnetic Beads and subjected to immunoblotting analysis.

### Single‐Cell Transcriptomic Analysis

Single‐cell transcriptomic data were extracted from the Gene Expression Omnibus (accession code GSE148673 and GSE131928), containing data from BRCA and GBM patients generated with the smart‐seq2 and 10X Genomics platforms. These data sets were integrated using Seurat's CCA to remove potential batch effects. Data processing and analysis were performed using Seurat (version 4.0, SCR_007322).

### RNA‐Seq

For samples sent for RNA‐Seq, total RNA was extracted using the Total RNA Extraction Reagent (Vazyme, R401‐01‐AA). RNA quality was further assessed on a 2100 expert Bioanalyzer and then total RNAs were sent for library preparation and sequencing on the Illumina Hisseq2000 platform by OE Biotech (Shanghai, China).

### Statistical Analysis

Three independent experiments were conducted and results were expressed as mean ± SD. Statistical normality and variance homogeneity were assessed, and significance was determined by Student's *t*‐test or one‐way ANOVA with Tukey's post hoc test. Mann–Whitney test was used when the data dispersion did not conform to the normal distribution. Survival rate was assessed by Mantel–Cox test. The Pearson correlation coefficient was used to assess the correlation between different data groups. For all statistical tests, P < .05 was considered significant. All data were expressed as the mean ± standard deviation (SD) values. Generally, all experiments were carried out with n ≥ 3 biological replicates. P < .05 was considered statistically significant. Analyses were performed using GraphPad Prism 8.0 software.

### Patient Consent Statement

BRCA surgical specimens and adjacent tissues were collected with the approval of the Institutional Ethics Review Boards of the First Affiliated Hospital of Nanjing Medical University in accordance with the guidelines of the International Ethical Guidelines for Biomedical Research Involving Human Subjects (CIOMS). Written informed consent was obtained from all patients (Ethics No.2021‐SR‐308)

## Conflict of Interest

The authors declare no conflict of interest.

## Author Contributions

K.Z. and T.S. contributed equally to this work. K.Z. and T.S. performed conceptualization. K.Z. and T.S. performed methodology. T.W., Z.C., J.Y., Q.S., L.L., Y.Z., and X.L. performed formal analysis and investigation. K.Z. and T.S. wrote and prepared the final manuscript. B.L. and N.L. wrote and reviewed and edited the final manuscript. D.Y., B.L. and N.L. acquired funding acquisition. L.L. and D.Y. performed resources. K.Z., B.L., and N.L. supervision. All authors read and approved the final manuscript.

## Supporting information



Supporting Information

## Data Availability

Data from RNA sequencing studies are available in the GEO database GSE17907, GSE23806, GSE24514 (Figure 1K), and GSE2990 (Figure 4B). All data generated or analyzed during this study are available from the authors. Further information and requests for resources and reagents should be directed to and will be fulfilled by the lead contact, Na Lu (nalu@cpu.edu.cn).
