# Supplementary material for: Nerve Growth Factor Signaling Promotes Nuclear Translocation of TRAF4 to Enhance Tumor Stemness and Metastatic Dormancy Via C‐Jun‐mediated IL‐8 Autocrine
Source: Adv Sci (Weinh). 2024 Dec 24;12(7):2414437. doi: 10.1002/advs.202414437 (PMC11831473; doi:10.1002/advs.202414437)
Supplement: Supplementary file 1 — Supporting Information [file ADVS-12-2414437-s001.docx]

**Table S1**


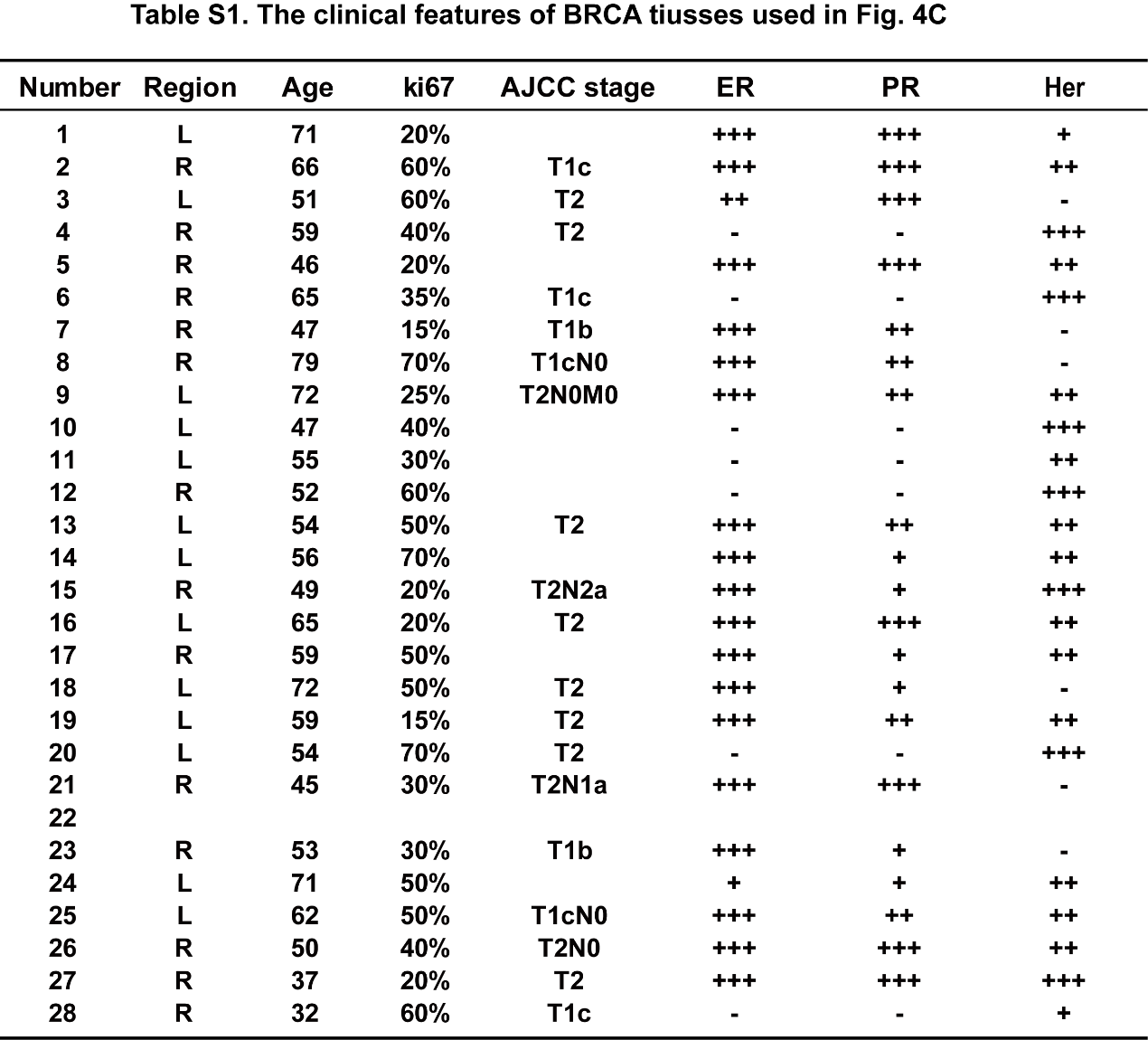


**Table S2**

**Primer and siRNA sequence:**

| myc-TRAF4-Full length | Forward | GGTACCATGCCTGGCTTCGACTACAAG |
| --- | --- | --- |
|  | Reverse | GAATTCTTACTTCCGGGGCAGTTCAACAGC |
| myc-TRAF4-Δring | Forward | TCAGCGGAAAAGCTTGGTACCGACTATGCCAAGATCTACCCAGACC |
|  | Reverse | TGCTGGATATCTGCAGAATTCTTACTTCCGGGGCAGTTCAA |
| myc-TRAF4-Δzinc finger | Forward-1 | CAGCGGAAAAGCTTGGTACCATGCCTGGCTTCGACTACAAGT |
|  | Reverse-1 | CTCACCAGGGCACACATCATGCGGATAGGCAGGCCCAA |
|  | Forward-2 | TATTGGGCCTGCCTATCCGCATGATGTGTGCCCTGGTGAGC |
|  | Reverse-2 | GCTGGATATCTGCAGAATTCTTACTTCCGGGGCAGTTCAA |
| myc-TRAF4-traf | Forward | TCAGCGGAAAAGCTTGGTACCGCCCTGGTGAGCCGGCAA |
|  | Reverse | AACGGGCCCTCTAGACTCGAGTTAGCTGAGGATCTTCCGGGG |
| myc-TRAF4-ring | Forward | GGTACCATGCCTGGCTTCGACTACAAG |
|  | Reverse | GAATTCTTACAGAGGAAGCTGGTCCTCAGG |
| myc-TRAF4-zinc finger | Forward | TCAGCGGAAAAGCTTGGTACCTGCATCCACAGTGAGGAGGG |
|  | Reverse | AACGGGCCCTCTAGACTCGAGTTAGGCCAGATGTGGCTTCAC |
| Myc-TRAF4-K11A | Forward | ACAAGTTCCTGGAGAGGCCCAAGCGACGGCTGCTG |
|  | Reverse | CCTCTCCAGGAACTTGTAGTCGAAGCCAGGCA |
| Myc-TRAF4-P12A | Forward | GTTCCTGGAGAAGGCCAAGCGACGGCT |
|  | Reverse | AGCCGTCGCTTGGCCTTCTCCAGGAAC |
| Myc-TRAF4-K13A | Forward | TTCCTGGAGAAGCCCAGGCGACGGCTGCTGTGCCC |
|  | Reverse | CTGGGCTTCTCCAGGAACTTGTAGTCGAAGCC |
| Myc-TRAF4-R14A | Forward | GGAGAAGCCCAAGGCACGGCTGCTGTGC |
|  | Reverse | GCACAGCAGCCGTGCCTTGGGCTTCTCC |
| Myc-TRAF4-R15A | Forward | CTGGAGAAGCCCAAGCGAGCGCTGCTGTGC |
|  | Reverse | GCACAGCAGCGCTCGCTTGGGCTTCTCCAG |
| GFP-TRAF4 | Forward | AGTCCGGACTCAGATCTCGAGCAATGCCTGGCTTCGACTACA |
|  | Reverse | GTACCGTCGACTGCAGAATTCTTACTTCCGGGGCAGTTCAA |
| Myc-TRAF4-NLS | Forward | TCAGCGGAAAAGCTTGGTACCCCAAAGAAGAAGCGGAAGGTCCCAAAGAAGAAGCGGAAGGTCCCAAAGAAGAAGCGGAAGGTCATGCCT |
|  | Reverse | TGCTGGATATCTGCAGAATTCTTACTTCCGGGGCAGTTCAA |
| Myc-TRAF4-P12A | Forward | ACCTCCATAGAAGATTCTAGAATGGCGGCGGAACAAAAA |
|  | Reverse | ATCCGATTTAAATTCGAATTCTTACTTCCGGGGCAGTTCAA |
| pGL3- CXCL8 Promoter | Forward | ATTTCTCTATCGATAGGTACCGGCCCAAGCTTTCTCTTCTTG |
|  | Reverse | ACTTAGATCGCAGATCTCGAGGCCTTATGGAGTGCTCCGG |
| pGL3- CXCL8 Promoter (+1--1712 bp) | Forward | ATTTCTCTATCGATAGGTACCTTTACTTACTTGTGTAACTAATTTTCCATATTC |
|  | Reverse | ACTTAGATCGCAGATCTCGAGGCCTTATGGAGTGCTCCGG |
| pGL3- CXCL8 Promoter (+1--1341 bp) | Forward | ATTTCTCTATCGATAGGTACCGCAAATTCACTGCTCTGTCGTACT |
|  | Reverse | ACTTAGATCGCAGATCTCGAGGCCTTATGGAGTGCTCCGG |
| pGL3- CXCL8 Promoter (+1--862 bp) | Forward | ATTTCTCTATCGATAGGTACCGAATGTGCTGTTCTCTTTCATCTTCC |
|  | Reverse | ACTTAGATCGCAGATCTCGAGGCCTTATGGAGTGCTCCGG |
| pGL3- CXCL8 Promoter (+1--400 bp) | Forward | ATTTCTCTATCGATAGGTACCATCTGTAATTAACTGAAAAAAAATAATTATGC |
|  | Reverse | ACTTAGATCGCAGATCTCGAGGCCTTATGGAGTGCTCCGG |
| Myc-TRAF4-Y6A | Forward | TTCGACGCCAAGTTCCTGGAGAAGCCCAAGCG |
|  | Reverse | GGAACTTTCGGTCGAAGCCAGGCATGAATTCC |
| Myc-TRAF4-Y166A | Forward | AGTGTCGCCTGTGAGAATAAGTGTGGTGCCCG |
|  | Reverse | TTCTCACAGCCGACACTCTCCTGGGGGCACAT |
| Myc-TRAF4-S209A | Forward | ATCCAGGCCCACCAGTACCAGTGCCCAAGGCT |
|  | Reverse | TACTGGTGggcCTGGATGGTGTCAAAGACGAACT |
| Myc-TRAF4-S212A | Forward | AGCCACCAGGCCCAGTGCCCAAGGCTGCCTGT |
|  | Reverse | CACTGGGCCTGGTGGCTCTGGATGGTGTCAAA |
| Myc-TRAF4-S242A | Forward | GAAGGACGCCTGTAACACCGCCCTGGTGCTCT |
|  | Reverse | TGTTACAGGCGTCCTTCAGATGGCCTGGCAGG |
| Myc-TRAF4-T422A | Forward | AGAAGCCAGGCGCGTGGCGGGGCTCCCTGGAT |
|  | Reverse | CCAcgcGCCTGGCTTCTGGAAATTCTTCCAGT |
| Myc-TRAF4-S426A | Forward | CGCCCTGGATGAGAGTTCTCTGGGCTTTGGTT |
|  | Reverse | AACTCTCATCCAGGGCGCCCCGCCACGTGCCTGG |
| Myc-TRAF4-S430A | Forward | TGGATGAGGCTTCTCTGGGCTTTGGTTATCCC |
|  | Reverse | CAGAGAAGCCTCATCCAGGGAGCCCCGCCACG |
| shTRAF4#1 | Forward | CGAAACTATGTGCGGGATGAT |
|  | Reverse | ATCATCCCGCACATAGTTTCG |
| shTRAF4#2 | Forward | CCGACCCAAACTGGAAGAATT |
|  | Reverse | AATTCTTCCAGTTTGGGTCGG |

**Table S3**

**Reagents:**

| Colchicine | Aladdin |
| --- | --- |
| Resveratrol | Aladdin |
| Ginsenoside Rg3 | Aladdin |
| Berberine | Aladdin |
| Paclitaxel | Aladdin |
| Quercetin | Aladdin |
| Genistein | Aladdin |
| Phloretin | Aladdin |
| Recombinant AKT1 | MCE |
| Recombinant GST-TRAF4 | Abnova |
| GSK2141795(AKTi) | Beyotime |
| FR180204(ERKi) | Beyotime |
| SP600125(JNKi) | Beyotime |
| rhIL-8 | Novoprotein |
| NGF | Novoprotein |
| PU139 | GLPBIO |
| PR619 | GLPBIO |
| MLN4924 | GLPBIO |
| LOXO-101 | GLPBIO |
| Reparixin | GLPBIO |

Oroxylin A (OA, C_16_H_12_O_5_) was supplied by the Professor Zhiyu Li's group of China Pharmaceutical University. Biotin-Oroxylin A (Biotin-OA, C_32_H_38_O_8_S) was synthesized and identified by the Professor Sun Haopeng's group of China Pharmaceutical University. These compounds were dissolved in DMSO (Merck, D1435) as a stock solution, stored at -20 °C, and diluted with the corresponding medium in each *in vitro* study. Preparation of Oroxylin A injection: 400 mg of arginine and 100 mg of Oroxylin A were added into 10 mL of saline, stirred at room temperature until the solution was clarified and transparent, and then filtered through 0.22 μm filter membrane and stored at 4 °C.

**Supplementary methods**

**Cells, cell culture and tissues**

Human colon cancer cell lines (HT29, SW480, Caco2, LS174T), human glioma cell lines (U-251 MG, T98G, A172) and human breast cancer cell lines (MCF7, MDA-MB-435S, BT549), human gastric cancer cell lines (MGC803, BGC823, MKN28, MKN45, SGC-7901), human normal breast Cell (MCF10A), human normal intestinal epithelial cells (NCM460), human normal astrocytes (HA1800) and human gastric mucosa cells (GES-1) were purchased from the Cell Bank of the Chinese Academy of Sciences (Shanghai, China). Murine breast cancer cell lines (67NR, 4TO7, 4T1) were a gift from professor Changying Guo’s lab in College of Biological Sciences, Xinjiang University. All cells were cultured in a medium supplemented with 10% FBS (Wisent, Nanjing, China). All cells were incubated at 5% CO2 at 37 °C. All cell lines were authenticated via STR profiling and were tested negative for mycoplasma contamination.

**Animal models**

MMTV-PyMT mouse model

Female FVB/NJGpt-Tg(MMTV-PyMT)/Gpt mice which develop spontaneous breast tumor, were purchased from GemPharmatech Co., Ltd(Nanjing, China).

**qRT-PCR Analysis**

Total RNA of cells and tissues were isolated by RNA-easy isolation reagent (Vazyme, Nanjing, China) and reverse transcript into cDNA using cDNA Synthesis Kit (Vazyme). ABI 7500 PCR machine (USA) was used to perform real-time quantitative PCR. The sequences of primers for human genes are as follows:

*Gapdh-* Forward*:* 5’-CAGCCTCAAGATCATCAGCA-3’;

*Gapdh-* Reverse*:* 5’-TGTGGTCATGAGTCCTTCCCA-3’

*IL-8-* Forward：5’-CCAAGCTTCCTTGTGCAAGTA-3’;

*IL-8-* Reverse：5’-AAGCCCAAAGTCCATCAGTGG-3’

*Sox9-* Forward*:* 5’-AGCGAACGCACATCAAGAC-3’;

*Sox9-* Reverse*:* 5’-CTGTAGGCGATCTGTTGGGG-3’

*Sox2-* Forward：5’-GCCGAGTGGAAACTTTTGTCG-3’;

*Sox2-* Reverse：5’-GGCAGCGTGTACTTATCCTTCT-3’

*Snail1-* Forward*:* 5’-TCGGAAGCCTAACTACAGCGA-3’;

*Snail1-* Reverse*:* 5’-AGATGAGCATTGGCAGCGAG-3’

*Slug-* Forward：5’-CGAACTGGACACACATACAGTG-3’;

*Slug-* Reverse：5’-CTGAGGATCTCTGGTTGTGGT-3’

**Detection of proteins bound by Bio-OA**

The cell lysates from tumor cells or HEK-293T cells, which have been transfected with Myc-TRAF4 plasmids for 24 h were collected and lysed and then centrifuged at 13000 rpm for 20 min. The supernatant was incubated with Bio-OA or OA for 3 h and then were analyzed by immunoblot and detected by Strep-HRP.

**Supplemental figures:**

**Fig.S1**


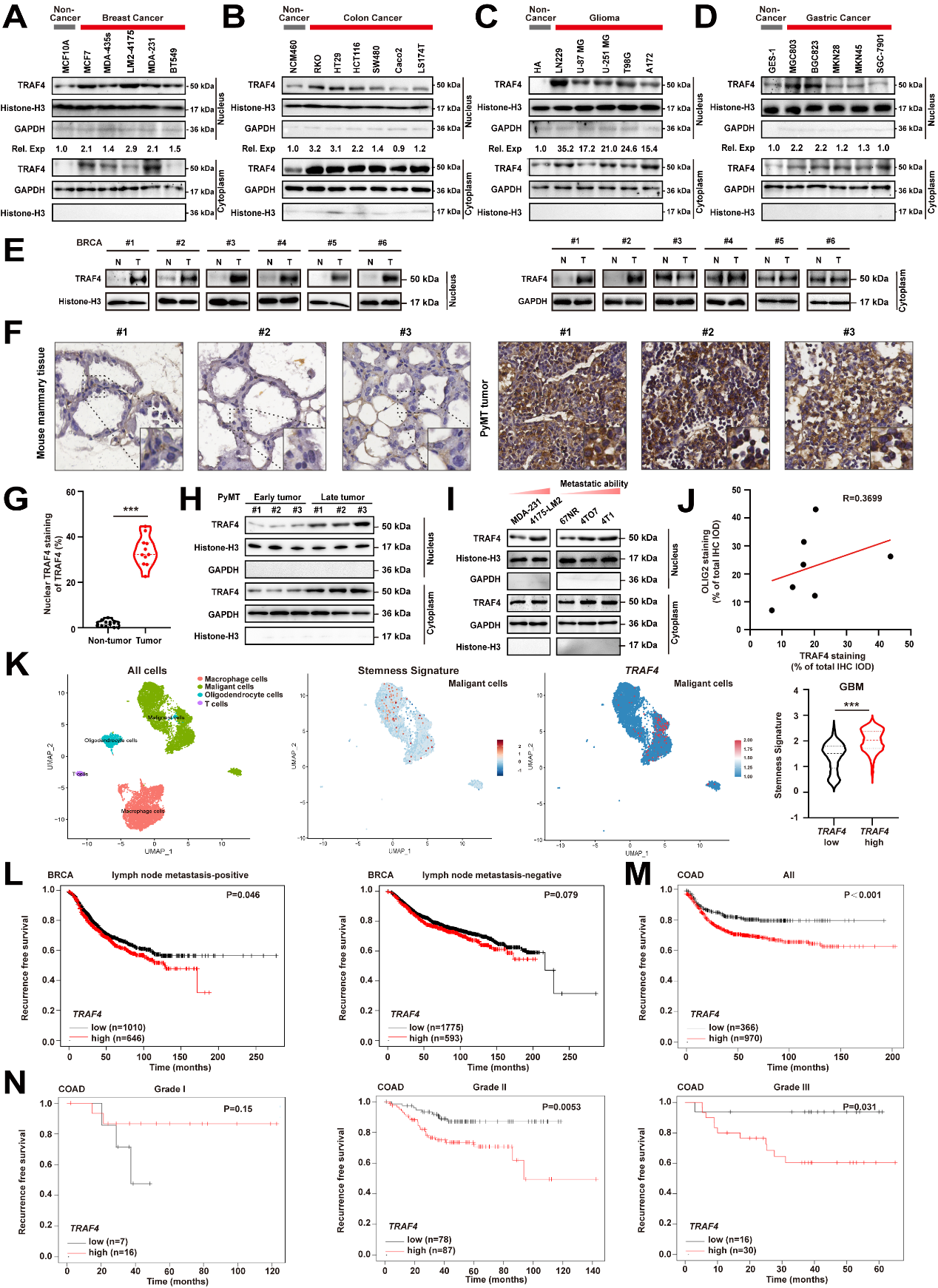
(A-D) Immunoblot analysis of TRAF4 expression in cytoplasm and nucleus in breast cancer cells, colon cancer cells, glioma cells, gastric cancer cells and their normal cells. Rel.Exp, relative expression of TRAF4 to Histone-H3 in the nucleus. Image J by comparing the expression of TRAF4 with the expression of Histone-H3 extracted within the image. (E) Immunoblot analysis of TRAF4 expression in cytoplasm and nucleus of paracancerous and tumor tissues in clinical samples from breast cancer patients (n = 6). (F) Representative IHC images (magnification×10, and ×20) of the TRAF4 staining (from three mice) in tumour tissues of MMTV-PyMT mice that spontaneously formed breast carcinomas and their mammary tissues; Bar, 100 μm. (G) Nuclear localization of TRAF4 is significantly increased in tumour tissues of MMTV-PyMT mice. Quantification of nuclear TRAF4 from MMTV-PyMT mice IHC tissue sections is shown. Staining (%) is calculated using Image J by comparing nuclear TRAF4 IHC integrated optical density (IOD) with total IOD values extracted within the image. Paired t-test. (H) Tumor tissues from MMTV-PyMT mice were collected and divided into two groups (early and late), and immunoblot analysis of TRAF4 expression in cytoplasmic and intranuclear tissues of tumours from two groups of mice. (I) Detection of nuclear TRAF4 expression of human-derived breast cancer cells and murine-derived breast cancer cells with different metastatic abilities by immunoblotting. (J) Correlation between TRAF4 and OLIG2 expression in BRCA tissues by using immunohistochemistry analysis of TRAF4 and OLIG2 expression. (K) Visualization of TRAF4 high and low cells in GBM tissue by UMAP (The samples of GEO DataSets, GSE131928). Visualization of TRAF4 and stemness signature genes expression (Runx1, Sox4, Sox9, Klf4, Klf9, Lgr4, Pou6f1) in GBM tissue by UMAP. Violin plot showing expression levels of stemness signature in TRAF4 high and low cells. The tips of the violin plot represent minima and maxima, and the width of violin plot shows the frequency distribution of data. (L)Kaplan–Meier analysis of the association between recurrence free survival and TRAF4 expression in patients with BRCA (lymph node metastasis-positive and lymph node metastasis-negative tissue). (M and N) Kaplan–Meier analysis of the association between recurrence free survival and TRAF4 expression in patients with COAD. The analyses for all COAD patients (above), Grade I BRCA patients (left), Grade II BRCA patients (middle) and Grade III BRCA patients (right) are shown. Data are presented as mean ± SD. *p<0.05, **p<0.01, ***p<0.001, n.s indicates non-significant.

**Fig.S2**


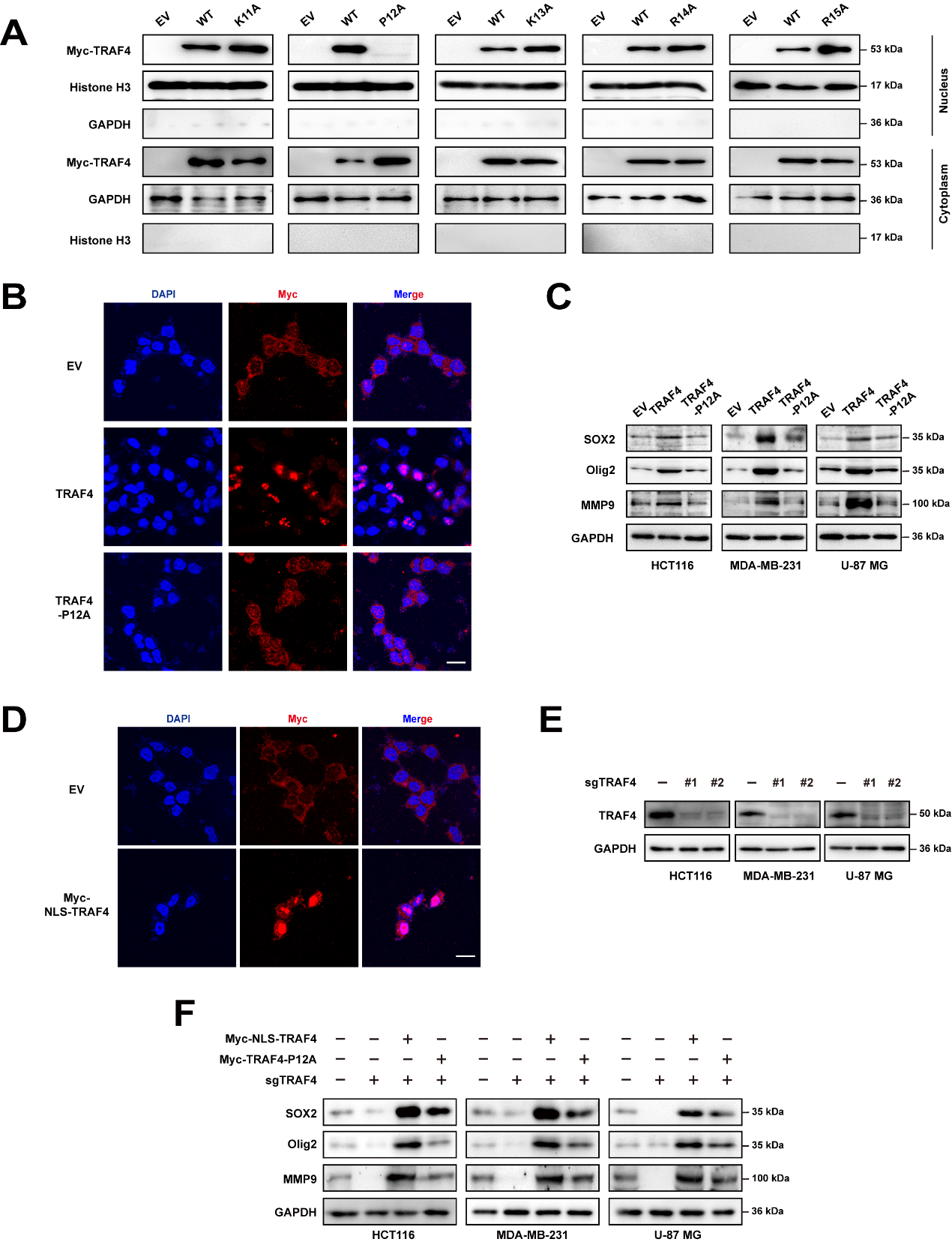


(A) Immunoblot analysis of nuclear TRAF4 expression in overexpressing TRAF4 plasmids with mutations in the nuclear translocation site. (B) IF images of nuclear TRAF4 expression in overexpressing TRAF4 and TRAF4-P12A tumor cells. Scale bar, 150 μm. (C) Immunoblotting analysis of SOX2, Oligo2, MMP9 and GAPDH in HCT116, MDA-MB-231, U-87 MG cells overexpressing EV, TRAF4 or TRAF4-P12A. (D) IF images of nuclear TRAF4 expression in overexpressing NLS-TRAF4 tumor cells. Scale bar, 150 μm. (E) Immunoblotting analysis of TRAF4 expression in HCT116, MDA-MB-231 and U-87 MG cells transfected with sgTRAF4#1, sgTRAF4#2. (F) TRAF4-KO HCT116, MDA-MB-231 and U-87 MG cells were overexpressed EV, TRAF4-P12A or NLS-TRAF4.

**Fig.S3**


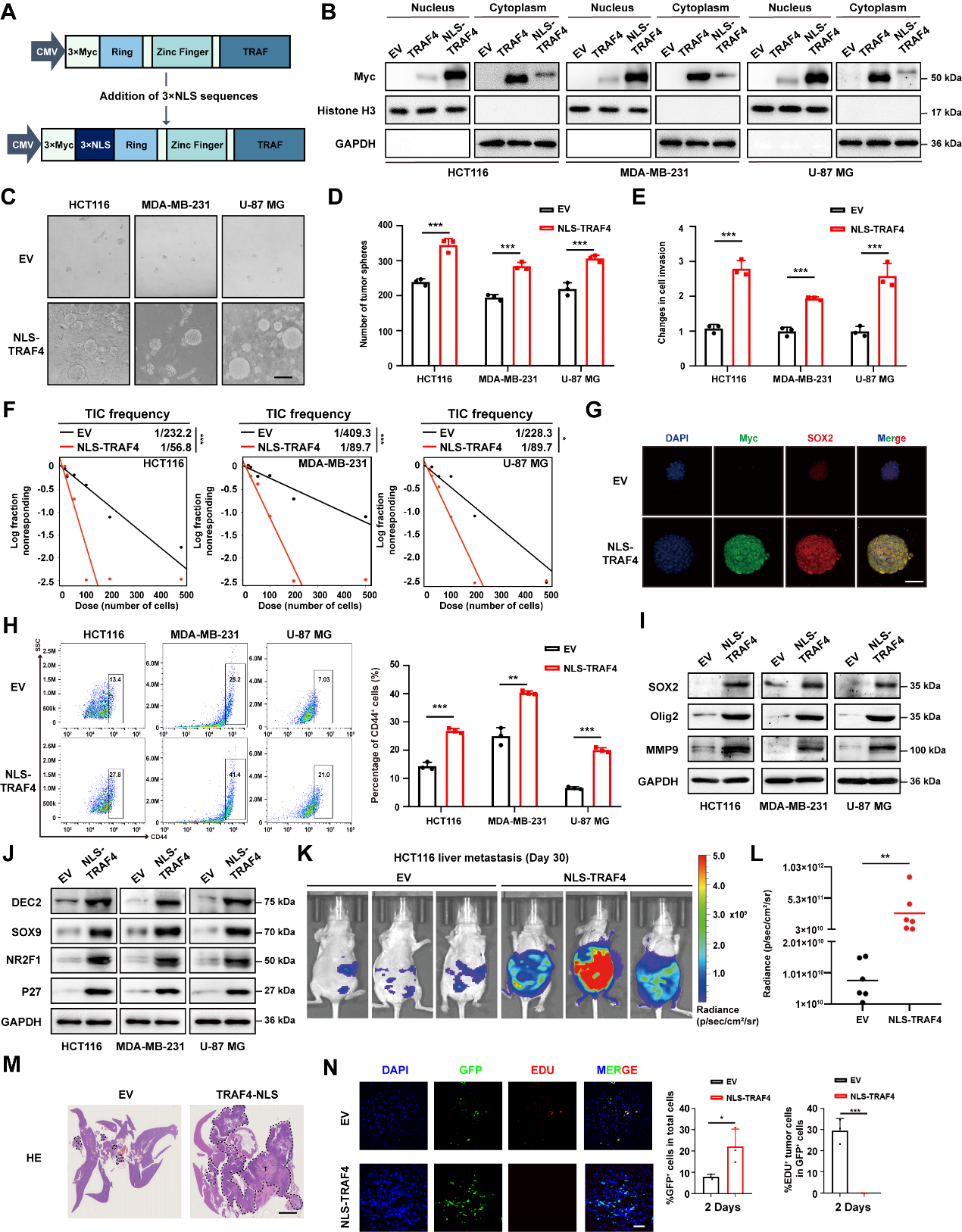


(A) Construction of TRAF4 plasmid containing 3× NLS nuclear localization sequence. (B) HCT116, MDA-MB-231 and U-87 MG cells were overexpressed EV, TRAF4 or NLS-TRAF4. (C and D) Representative images of tumorspheres overexpressed EV or NLS-TRAF4 and the number of tumorspheres overexpressed EV or NLS-TRAF4. Scale bar, 25 μm. (E) The quantification of cell invasion of HCT116, MDA-MB-231, U-87 MG cells overexpressing EV or NLS-TRAF4. (F) Frequency of tumor sphere formation was tested by *in vitro* limiting dilution assay in HCT116, MDA-MB-231, U-87 MG cells overexpressing EV or NLS-TRAF4. (G) IF images of expression of Myc-TRAF4 (green) and SOX2(red) in HCT116 tumorsphere. Scale bar, 150 μm. (H) CD44 expression was detected in HCT116, MDA-MB-231, U-87 MG cells overexpressing EV or NLS-TRAF4. Right side is its quantification. (I) Immunoblotting analysis of SOX2, Oligo2, MMP9 and GAPDH in HCT116, MDA-MB-231, U-87 MG cells overexpressing EV or NLS-TRAF4. (J and K) A total of 2 × 10^6^ HCT116 cells overexpressing EV or NLS-TRAF4 were injected into the spleen to establish the colorectal cancer liver metastasis model. After 30 days, Bioluminescence was detected(J). Quantification of Bioluminescence(K) is shown. (L)Representative images of H&E staining from two groups of mice. Scale bar, 10 μm. (M) Seeding and proliferation of HCT116 overexpressing EV or NLS-TRAF4 subpopulations in livers at 2 days after injected into the spleen Shown are representative immunofluorescences (IF) images, a percentage of GFP^+^ tumor cells in total cells (middle) and EdU^+^ proportions of tumor cells (right); *n* = RMFs from 3 mice. Scale bar, 150 μm. Each experiment was performed at least three times. Data are presented as mean ± SD. *p<0.05, **p<0.01, ***p<0.001, n.s indicates non-significant.

**Fig.S4**


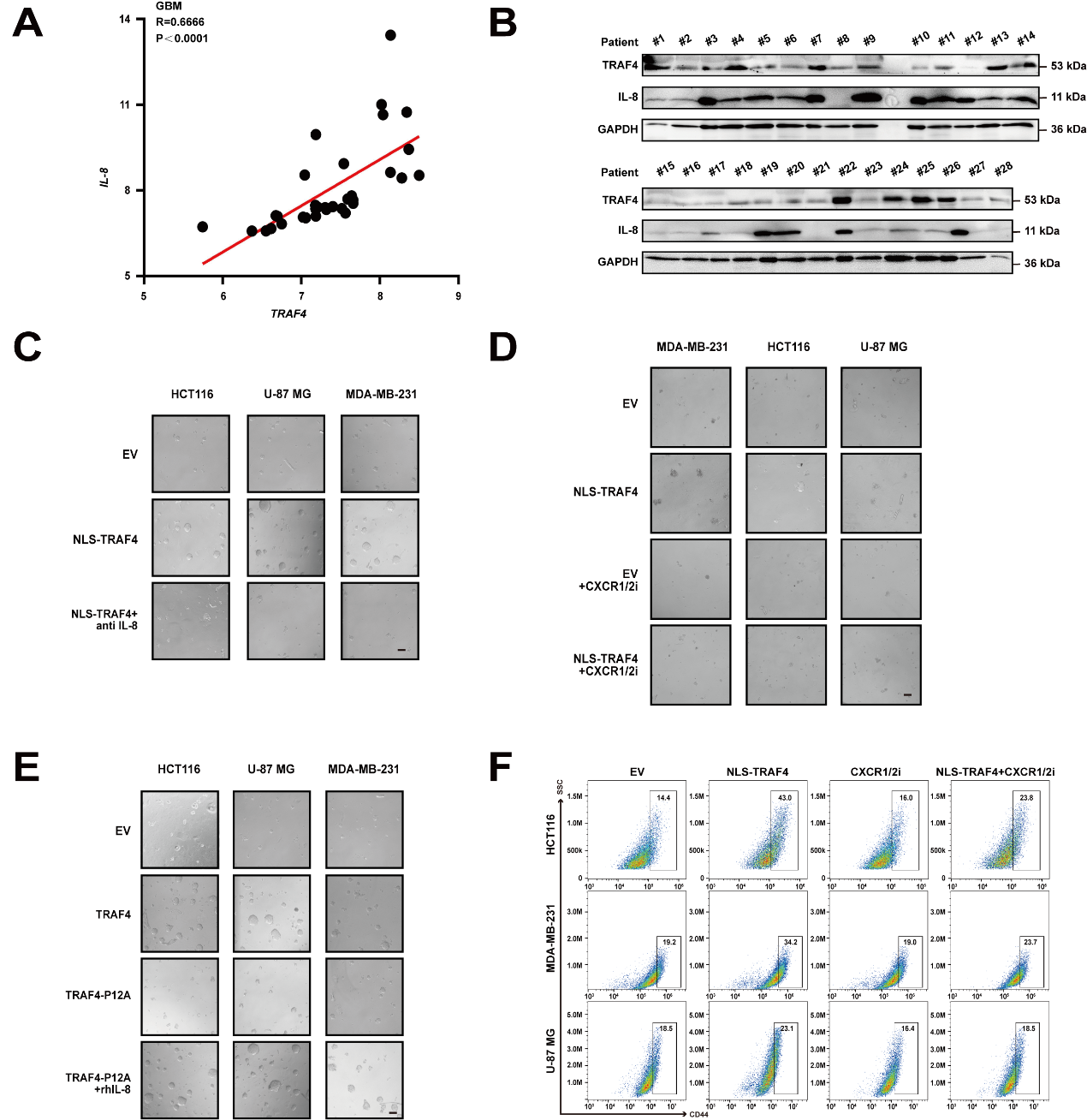


(A) Correlation between *TRAF4* and *IL-8* expression in GBM tissues by using GEO dataset of GSE131928. (B) Immunoblot analysis of nuclear and cytoplasmic TRAF4 and IL-8 expression in BRCA tissues (n = 28). (C) Representative images of tumorspheres overexpressing EV, NLS-TRAF4 or NLS-TRAF4 + anti-IL-8(500 ng/mL). (D) Representative images of tumorspheres overexpressing EV, NLS-TRAF4 or NLS-TRAF4 + CXCR1/2i (Reparixin, 100 nM) (E) Representative images of tumorspheres overexpressing EV, TRAF4, TRAF4-P12A or TRAF4-P12A + rhIL-8 (Recombinant human IL-8 protein, 200 ng/mL). (F) Flow cytometry analysis of CD44^+^ cell ratio in different groups of HCT116, MDA-MB-231 and U-87 MG cells.

**Fig.S5**

**
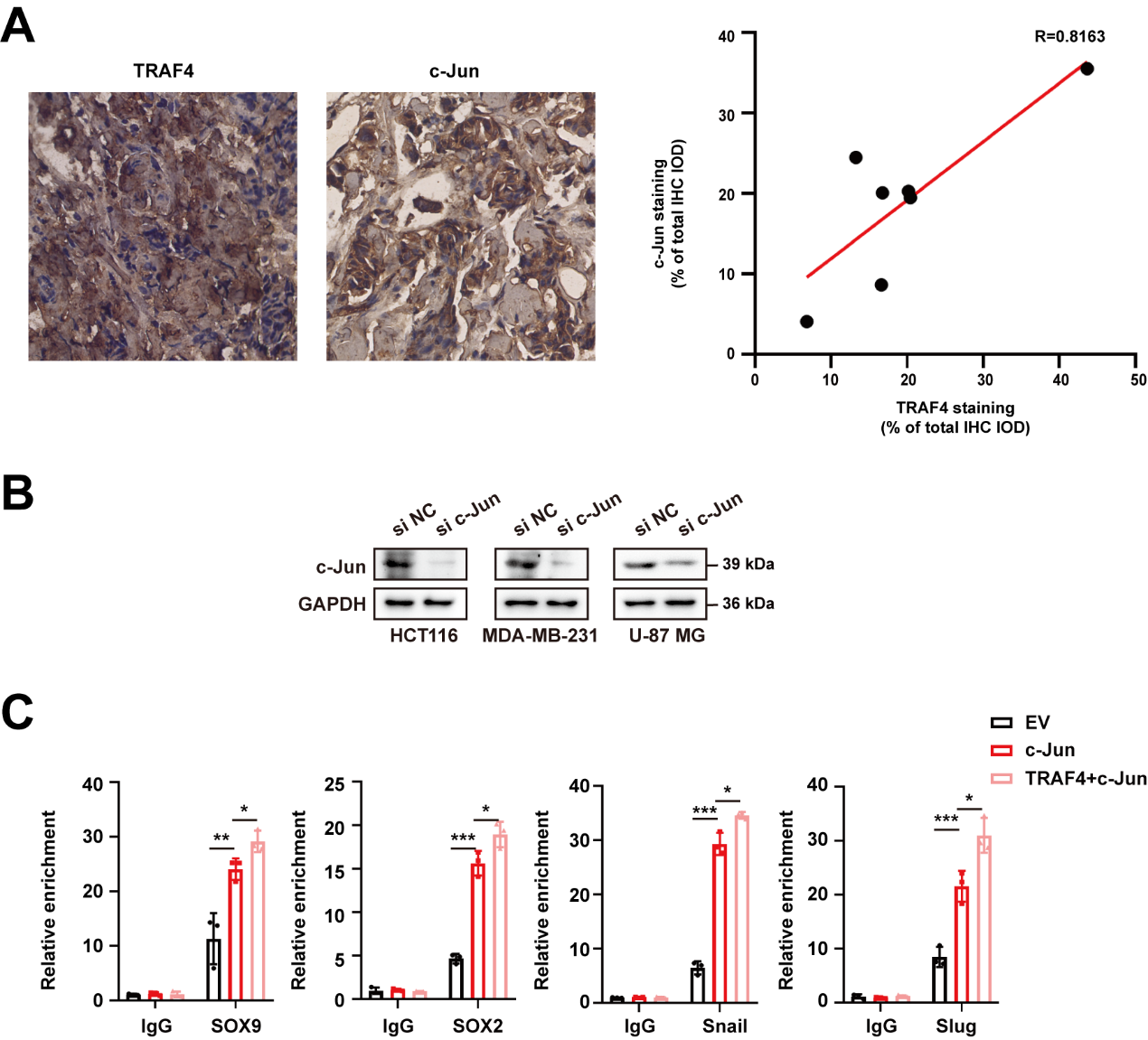
**

(A) Correlation between TRAF4 and c-Jun expression in BRCA tissues by using immunohistochemistry analysis of TRAF4 and c-Jun expression. (B) Immunoblotting analysis of c-Jun expression in HCT116, MDA-MB-231 and U-87 MG cells transfected with c-Jun. (C) Chromatin from HCT116 cells transient expressing c-Jun or TRAF4+c-Jun were analyzed for recruitment of *Sox9, Sox2, Snail1, Slug* by ChIP-qPCR. n = 3 biologically independent samples, Student’s t-test.

**Fig.S6**


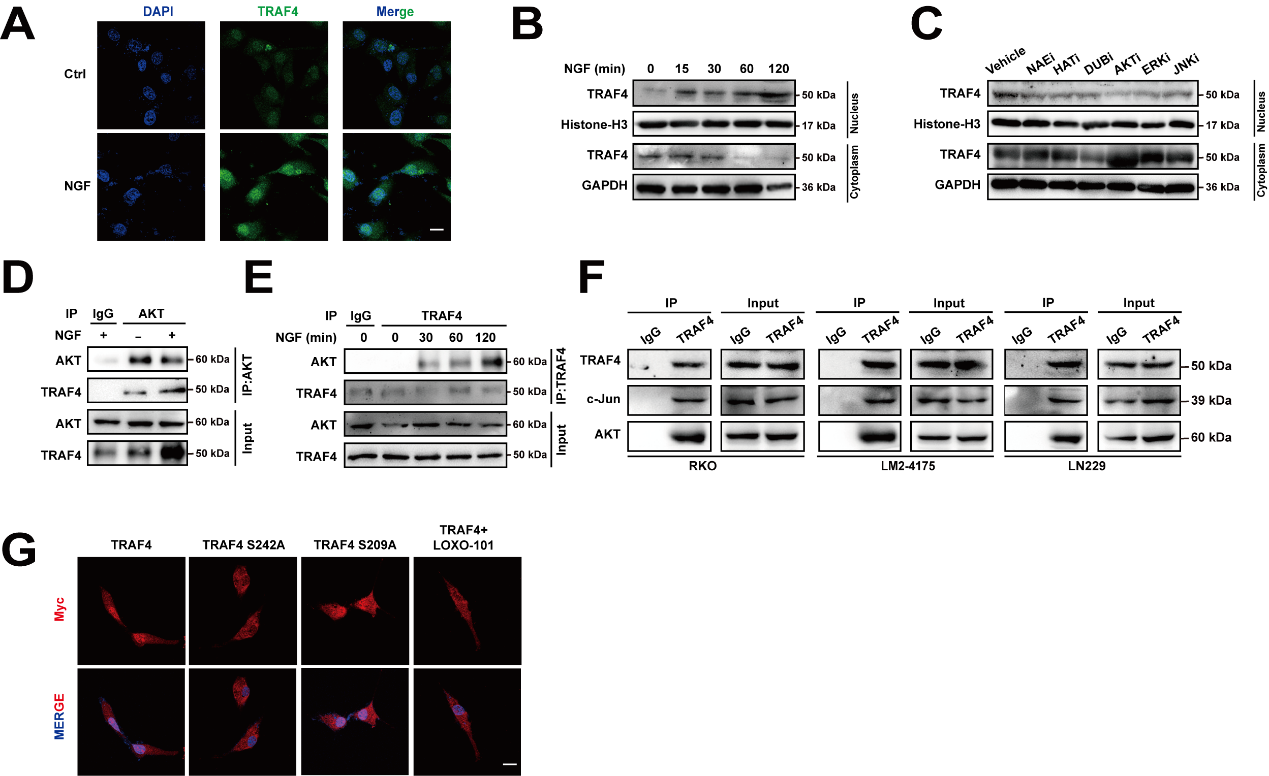


(A) NGF treatment promotes TRAF4 nuclear translocation. U-87 MG cells were treated with or without NGF for 3 h and TRAF4 subcellular localization was examined via IFA assay. Scale bar, 150 μm. (B) Time-dependent analysis of nuclear TRAF4 by immunoblotting after NGF treatment in U-87 MG cells. (C) Expression of nuclear TRAF4 in U-87 MG cells after administration of protein post-translational modification inhibitors detected by immunoblotting. (D) AKT interacts with TRAF4. U-87 MG cells treated with NGF for 3 h were subjected to AKT immunoprecipitation. (E) NGF treatment promotes TRAF4–AKT interaction. U-87 MG cells were treated with NGF for indicated time points followed by TRAF4 Co-IP assay. (F) AKT and c-Jun interact with TRAF4. RKO, LM2-4175 and LN229 cells were subjected to TRAF4 immunoprecipitation. (G) pTRAF4^S242^ enhances the nuclear localization of TRAF4. U-87 MG cells were transfected with indicated plasmids for 24 h before processing for IFA assay. LOXO-101, a selective inhibitor of the tropomyosin-related kinase.

**Fig.S7
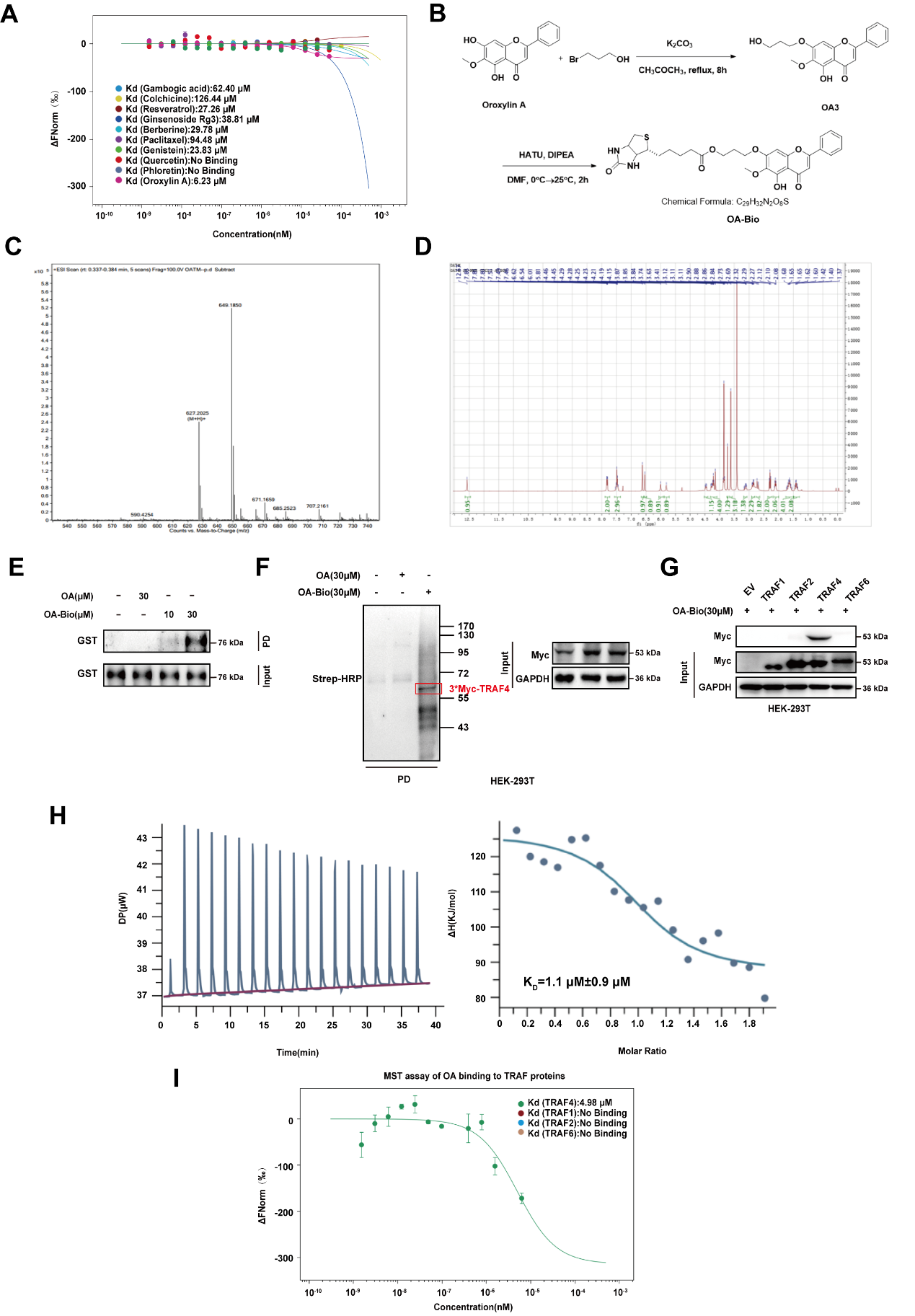
**

(A) MST assay was peformed to screen for the best natural product binding to TRAF4 from 10 natural products. (B) The synthesis route of OA-Bio. (C) NMR analysis of OA-Bio. (D) Mass spectrometry of OA-Bio. (E) Purified human GST-TRAF4 protein was incubated with indicated doses of OA-Bio (10 μM, 30 μM) and then pulled down using streptavidin beads. (F) HEK-293T cell lysates overexpressing Myc-TRAF4 were incubated with OA-Bio or OA and detected by Strep-HRP. (G) HEK-293T cell lysates overexpressing Myc-TRAF1, TRAF2, TRAF3, TRAF4 were incubated with OA-Bio, which were then pulled down using streptavidin beads. (H) ITC assay was peformed for the affinity between OA and purified GST-TRAF4 protein. (I) MST assay for the affinity between OA and purified GFP-TRAF4/GFP-TRAF1/GFP-TRAF2/GFP-TRAF6 protein.
